# Unlocking the potential of titanium dioxide nanoparticles: an insight into green synthesis, optimizations, characterizations, and multifunctional applications

**DOI:** 10.1186/s12934-024-02609-5

**Published:** 2024-12-23

**Authors:** Ahmed Ghareeb, Amr Fouda, Rania M. Kishk, Waleed M. El Kazzaz

**Affiliations:** 1 Botany and Microbiology Department, Faculty of Science, Suez Canal University, Ismailia, 41522 Egypt; 2 Botany and Microbiology Department, Faculty of Science, Al-Azhar University, Nasr City, Cairo 11884 Egypt; 3 Microbiology and Immunology Department, Faculty of Medicine, Suez Canal University, Ismailia, 41522 Egypt

**Keywords:** TiO_2_-NPs, Biogenic synthesis, Biomedical applications, Targeted drug delivery, Bioimaging, Biosensors, Agricultural applications, And environmental remediation

## Abstract

This comprehensive review explores the emergence of titanium dioxide nanoparticles (TiO_2_-NPs) as versatile nanomaterials, particularly exploring their biogenic synthesis methods through different biological entities such as plants, bacteria, fungi, viruses, and algae. These biological entities provide eco-friendly, cost-effective, biocompatible, and rapid methods for TiO_2_-NP synthesis to overcome the disadvantages of traditional approaches. TiO_2_-NPs have distinctive properties, including high surface area, stability, UV protection, and photocatalytic activity, which enable diverse applications. Through detailed analysis, this review demonstrates significant applications of green fabricated TiO_2_-NPs in biomedicine, explicitly highlighting their antimicrobial, anticancer, and antioxidant activities, along with applications in targeted drug delivery, photodynamic therapy, and theragnostic cancer treatment. Additionally, the review underscores their pivotal significance in biosensors, bioimaging, and agricultural applications such as nanopesticides and nanofertilizers. Also, this review proves valuable incorporation of TiO_2_-NPs in the treatment of contaminated soil and water with various environmental contaminants such as dyes, heavy metals, radionuclides, agricultural effluents, and pathogens. These comprehensive findings establish the foundation for future innovations in nanotechnology, underscoring the importance of further investigating bio-based synthetic approaches and bioactivity mechanisms to enhance their efficacy and safety across healthcare, agricultural, and environmental applications.

## Introduction

In the 1980s, nanotechnology breakthroughs allowed scientists to manipulate atomic particles, enabling the engineering of nanoscale materials and structures. This innovation has applications in biomaterials, organic chemistry, and medicine [1]. This microscopy technology has reshaped modern medicine, introducing new approaches to drug delivery, body imaging, and disease detection with remarkable precision [2]. Nanotechnology has progressed challenging diagnoses and advanced understanding of disease mechanisms. Due to their molecular-level size, nanoparticles enhance treatments in both in-vivo and in-vitro lab settings. These microscopic carriers facilitated targeted drug delivery in high-risk areas, delivering exact doses while minimizing side effects [3].

Nanoparticles (NPs) possess significant advantages compared to bulk materials, including expanded surface area, elevated surface energy, constrained nano environment, and decreased imperfections [4]. Characteristics of nanoparticles, like size, shape, chemical makeup, surface architecture, and crystallinity, heavily influence their potential uses across many areas, including pharmacological domains such as antimicrobial [5, 6], antioxidant [7], anticancer [8], anti-tyrosinase potential [9] as well as industrial and optoelectronics domains [10].

In this regard, transition metals have garnered significant attention due to two key factors: firstly, their atomic electronic configuration features incomplete d-orbitals, and secondly, their ability to undergo fluctuating oxidation phases. Consequently, their intrinsic physicochemical properties can be tailored for a vast array of applications across the fields of natural sciences and materials engineering [11]. Furthermore, these metallic elements can generate a diverse range of oxide compounds, which present promising prospects for expanding the frontiers of research across burgeoning domains such as environmental studies, agricultural applications, medical advancements, cosmetic formulations, energy storage solutions, fuel cell technologies, semiconductor device development, sensor innovations, and catalytic processes [12]. Particle size is a pivotal factor that significantly influences the fundamental properties of any material. Firstly, with regard to structural properties, bulk oxides exhibit robustness and low phase stability, whereas nanoparticle oxides can readily undergo structural transformations and possess low surface free energy due to their reduced particle size [13]. Secondly, concerning electronic properties, the surfaces of bulk oxides are extended due to the distribution of ionic charge as compared to the nanoscale structure [14]. Thirdly, the band gap of bulk oxides is broad. In contrast, NP oxides exhibit a low band gap, enhancing conductivity and chemical reactivity [15]. Consequently, by modulating the particle size, materials’ structural, electronic, physical, and chemical properties can be tailored to meet specific requirements across various applications. Therefore, as a result, oxide nanoparticles, owing to their size limitations and abundant density of corner surface sites, possess the ability to exhibit unique physical and chemical properties [16].

Due to the high production of titanium oxide nanoparticles (TiO_2_-NPs), approximately 10 × 10^3^ tons in 2011, it is considered the major and promising industrial substance [17]. TiO_2_-NPs owe their widespread use to their economical production costs, superior chemical stability, elevated refractive index, robust oxidation capabilities, and the existence of oxygen vacancies in their crystalline lattice [18]. The substantial band gap exhibited by TiO_2_-NPs is a pivotal property that renders them suitable for semiconductor applications in the optical industry [19]. Attributed to the exceptional electrical and ionic properties of TiO_2_-NP_S_, these materials can be further customized and tailored to be utilized in the fields of sensor technology and electronic device fabrication [20].

TiO_2_-NP_S_ manifests as a white, water-insoluble powder with a remarkably high refractive index of 2.4, rendering it suitable for pigments in the paint industry [21]. Notably, TiO_2_ naturally occurs in three distinct polymorphic forms - rutile, anatase, and brookite - each possessing a crystalline structure. These polymorphs find extensive applications in the gemstone industry [22]. Furthermore, owing to their unique crystalline nature, the physical and chemical properties of TiO_2_-NPs can be tailored by modifying the ratio of these polymorphic forms, thereby expanding their scope of applications across various sectors. The remarkable versatility of TiO_2_-NPs is highlighted by their extensive and successful applications across diverse domains. These nanoparticles have proven invaluable in the field of sensors [23]. While also serving as photocatalysts for the decomposition of wastewater pollutants [24]. Furthermore, their antimicrobial and antibacterial properties [25] allow for their use as food additives and in cosmetic products [26].

In this review, we delve into different eco-friendly, safe, and sustainable synthesis techniques for TiO_2_-NPs production. This eco-friendly approach offers significant advantages over conventional industrial methods, notably improved reagent handling and enhanced process safety [27]. Furthermore, to the best of our knowledge, we have endeavored to provide a comprehensive summary of the various biomedical applications of TiO_2_-NPs, encompassing their antioxidant, antibacterial, antifungal, antiviral, and anticancer with other biotechnological and environmental applications. This review aims to elucidate our current understanding within this field and inspire the exploration of more sophisticated nanostructures in the forthcoming years. By elucidating the synthesis methods and potential applications, we strive to pave the way for future advancements and innovations in the realm of TiO_2_-NPs.

## Synthesis methods

### Conventional methods

Conventional methods for synthesizing metal oxides can be broadly categorized into two distinct approaches: top-down and bottom-up. The top-down approach involves breaking down bulk macroscopic particles into nanoscopic particles (1 mm-xµ) through various physical methods. This approach is relatively straightforward than the bottom-up but also encounters monodispersed challenges and limited particle control. Various physical techniques involved, such as milling, etching, sputtering, pulse laser ablation, and evaporation-condensation techniques [28].

On the other hand, the bottom-up approach relies on a self-assembly process, whereby atomic nuclei are joined together to form nanosized particles (0.1 nm-xµ). This approach facilitates the easy manipulation of nanoparticle dimensions and morphology, resulting in superior homogeneity. However, its drawbacks include scalability, strict resource constraints, and multi-phase implementation. It encompasses several techniques and processes, including chemical vapor deposition, sol-gel processes, hydrothermal methods, sonochemical techniques, flame spraying, spinning, and green synthesis methods [29]. Notably, while the top-down approach focuses on reducing the size of bulk materials to the nanoscale, the bottom-up approach builds nanoparticles from the ground up, starting with atomic or molecular precursors [30]. The choice between these approaches depends on several factors, such as the desired material properties, cost-effectiveness, and scalability requirements.

Researchers have developed a wide range of chemical and physical approaches to synthesize nanoparticles with diverse geometries, enabling their application across numerous fields. Among the novel techniques employed for achieving these distinct nanoparticle geometries, a clear distinction can be drawn between lithography-based techniques and non-lithographic methods [31]. The lithography techniques, which include photolithography, ion beam lithography, microcontact printing, dip pen lithography, and nanoimprint lithography, involve patterning materials on a surface through various lithographic processes [32]. These techniques allow for precise control over the geometric features of the nanoparticles. On the other hand, non-lithographic techniques encompass methods such as ball milling, a mechanical process for grinding and mixing materials, evaporation-condensation, and electrochemical synthesis, which utilizes electrochemical reactions to synthesize nanoparticles [33]. These non-lithographic approaches offer alternative routes for nanoparticle synthesis, potentially enabling unique geometric configurations.

Nonetheless, the methods mentioned earlier frequently necessitate multiple processing steps, stringent control over variables such as pressure, pH, and temperature, and the employment of expensive equipment and toxic chemicals. Furthermore, these techniques often result in the generation of toxic by-products that pose a significant threat to ecosystems [34]. In light of these challenges, there is an urgent need to develop eco-friendly approaches utilizing biological and green synthesis techniques.

### Biogenic synthesis

Considering a broader perspective, biological approaches embrace the concept of “green synthesis” for the production of nanoparticles [35]. This method can be further categorized into two distinct pathways: (a) phytosynthesis, where the synthesis process is facilitated by leveraging the capabilities of plants and their extracted compounds, and (b) microbial synthesis, which involves harnessing the synthetic potential of microorganisms such as bacteria, algae, fungi, yeasts, and actinomycetes, utilizing extracts derived from these organisms. The phytosynthetic route exploits the natural ability of plants to synthesize materials by harnessing their varied metabolites. At the same time, the microbial pathway capitalizes on the metabolic machinery of various microbial species to generate the desired metal and its oxides-nanoparticles. These biological methods offer an environmentally friendly and sustainable alternative to conventional synthesis techniques [36, 37].

Green synthesis surpasses conventional processes by integrating non-toxic biological organisms, natural acids, and water as solvents and safer catalysts while replacing hazardous chemical substances. This eco-green strategy significantly enhances reaction efficiency with faster synthesis times and higher yields for industrial scalability (Table 1). Concurrently, it generates substantial product yields of biocompatible nanoparticles that are imperative for use within the medical and pharmaceutical domains [38].

The green synthesis approach faces several key challenges that can potentially obstruct its successful implementation. Firstly, the optimization processes required to synthesize nanoparticles with specific size distributions and morphological characteristics are inherently tied to their intended biological functions (Table 1). Secondly, deciphering the distinct role played by each constituent compound involved in the biofabrication process mandates a comprehensive chemical analysis of the filtered biological biomass [39].

**Table 1:** Summarizing the advantages and disadvantages of biogenic NPs synthesis

Biogenic NPs synthesis	Advantages	Disadvantages	Reference
**Plant-Derived NPs**	- Bio-safe, inexpensive, and quick Production process - Eco-sustainable approach (contaminants and pollutants recycling) - Optimize synthesis oversight with stable and uniform NPs yield - Biomass waste utilization and reusability	- Genetically unmodifiable, unlike microorganisms	[35, 36, 38, 40]
**Microbial Mediated NPs**	- Decreased requirement for extra capping or stabilizing agents. - Can be genetically altered for higher yields. - Less handling of toxic chemicals & lower infrastructure requirements - Quick and easy to adjust and manipulate synthesis parameters (PH, temp.etc.) - Adaptive capabilities with different synthesis routes (intracellular, extracellular, and cell-free extract synthesis. - Cost and energy-efficient approach - Versatile biomedical and industrial applications	- Scaling up manufacturing for commercial production (contamination, sterility, and cost concern). - Multiple separation steps are needed, as well as batch-to-batch yield variation with the possibility of NPs aggregation over time - Bioproduct quality variance. - Constrained dimensional control and NPs instability for particular NPs	

#### Phytosynthetic routes

The synthesis of nanoparticles facilitated by plants is considered more stable compared to the synthesis mediated by microbes. The process of producing nanoparticles using plant extracts is straightforward, economical, and yields a high quantity while adhering to sustainable practices [41]. These plant extracts can be derived from various plant parts, such as flowers, roots, seeds, or leaves. Among the different plant parts, leaves are more commonly utilized for obtaining extracts, as they are abundant in metabolites [42]. Leaves offer a more viable option for deriving extracts without generating toxic byproducts. These processes involving leaf extracts are straightforward, economical, and non-toxic, making them easily accessible and compatible for NPs production with appropriate quantities suitable for industrial and environmental sectors [43].

Significantly, plant extracts are abundant in polyphenolic compounds that function as potent reducing agents, thereby facilitating the reduction of metal ions and consequently leading to the formation of nanoparticles [44]. Additionally, the reduction of these metallic ions can be prompted by various cellular components such as amines, carbonyls, phenolic compounds, pigments, terpenoids, and alkaloids [45]. This synthesis process is driven by redox reactions involving metallic ions and secreted molecules like sugars, carbohydrates, and proteins. However, elucidating the precise mechanism of action remains challenging due to the vast chemical diversity of the metabolites involved in the reduction process [46]. Nevertheless, experimental parameters such as pH, temperature, reactant concentration, and reaction time play a pivotal role in determining the physical-chemical properties of the resultant nanoparticles [47]. Moreover, the biodiversity and availability of different plant families, each with unique profiles of primary and secondary metabolites, contribute significantly to producing various biogenic nanoparticles with important antimicrobial applications [48].

**Fig. 1:**
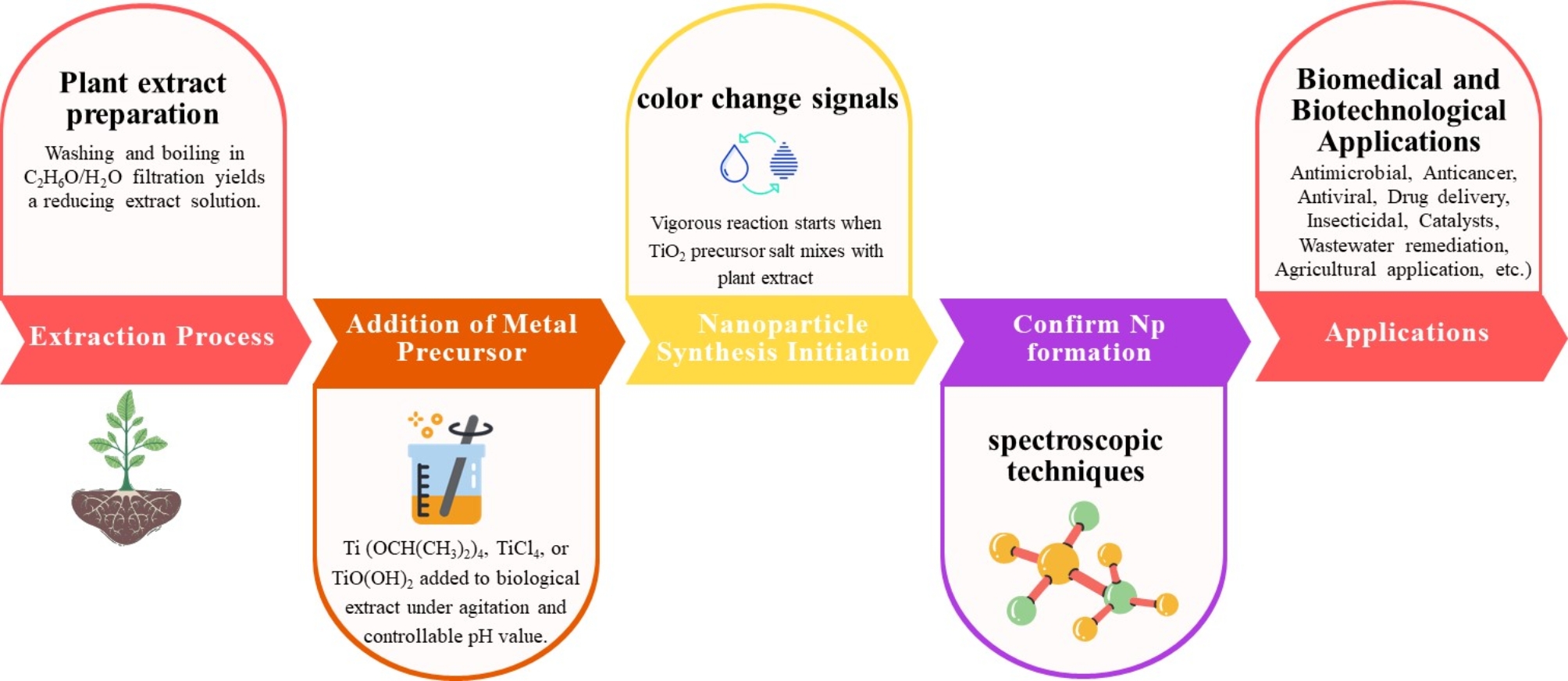
Flowchart showing plant-mediated production of TiO_2_-NPs starting from plant extract to applications

The desired plant part undergoes thorough washing and cleaning, followed by boiling in a solvent like ethanol (C_2_H_6_O) or dH_2_O and subsequent filtration (Fig. 1). The resultant filtered solution, rich in plant extracts, serves as a reducing agent. To this filtered solution, a suitable metallic or metallic oxide precursor such as titanium tetraisopropoxide (Ti(OCH(CH_3_)_2_)_4_), titanium tetrachloride (TiCl_4_), or titanyl hydroxide (TiO(OH)_2_) for TiO_2_-NPs synthesis is introduced under constant agitation [49]. The reaction mixture starts vigorously when the TiO_2_ precursor salt is combined with the plant extract, and a color change indicates the first sign of nanoparticle synthesis [50]. This initial observation can then be confirmed afterward by spectroscopic techniques, leading to the formation and characterization of the desired TiO_2_-Nps [51].

A major portion of green synthesis studies utilize leaf extracts, as leaves are abundant reservoirs of metabolites. For example, Extracts from *Moringa oleifera* leaves facilitated the synthesis of TiO_2_-NPs around 100 nm in size with varying shapes, exhibiting promising wound healing capabilities [52]. Another TiO_2_-NP was synthesized using the leaf extract of *Hibiscus rosasinensis* L. and exhibited potent antimicrobial activity against both Gram-negative and Gram-positive bacterial strains [53]. In another study, TiO_2_-NPs derived from *Nyctanthes* leaf extracts possessed a uniform spherical shape and ranged in size from approximately 100 to 150 nm. These biogenic NPs demonstrated significant pediculicidal (lice-killing), acaricidal (mite-killing), and larvicidal (larva-killing) properties [54]. Similarly, the leaf extract of *Calotropis gigantea* L. was reported to facilitate the reduction of TiO_2_ to nanoparticles within a span of 6 h, attributed to the presence of primary amines in the extract. The bio-mediated TiO_2_-NPs exhibited promising acaricidal activity against the larvae of *Rhipicephalus microplus* and *Haemaphysalis bispinosa* [55].

The synthesis of TiO_2_-NPs has also been reported using the aqueous leaf extract of the medicinal plant *Catharanthus roseus* (L.). The aliphatic alcohols and amines present in the extract contributed to the formation of TiO_2_-NPs with irregular morphologies and particle sizes ranging between 25 and 110 nm [56]. Notably, the variation in size and morphology observed among the reported studies is influenced by factors such as reaction temperature, time, and the source of the plant extract [57]. Thus, optimizing these parameters can lead to improvements in the synthesis mechanism [58]. Furthermore, a wide array of propitious plant extracts remains to be explored for synthesizing TiO_2_-NPs. Table 2 shows the green synthesis of TiO_2_-NPs using plant extracts regarding their sizes, shapes, and applications.

**Table 2:** Examples of some plants-derived TiO_2_-NPs and their applications

Plant Species	Size	Shape	Applications / Properties Studied	Reference
*Morus nigra* (Mulberry)	0.17 nm (TiO_2_(Aq)), 0.076 nm (TiO_2_(Et))	round shape- irregular surface morphologies	Antimicrobial activity against *Staphylococcus aureus* and *Proteus mirabilis*	[59]
*Carthamus tinctorius* (Safflower)	47 nm	Spherical, irregular	Alternative fuel additives for diesel-biodiesel blends, impact on engine performance and emissions	[60]
*Phyllanthus niruri*			Industrial wastewater treatment	[61]
Ivy leaf extract	26.34 nm	irregular spherical and tetragonal shape	Cefoperazone removal from pharmaceutical wastewater, antimicrobial activity	[62]
*Punica granatum* (Pomegranate)	100–150 nm	spherical	Antibacterial activity against *E. coli*	[63]
*Mentha arvensis*	80.51–200 nm	Triangular aggregation pattern	Antioxidant activity (DPPH, FRAP assays)	[64]
*Mangifera indica* (Mango) *Azadirachta indica* (Neem)	3–27 nm 8–28 nm	agglomerated spherical-like morphology less agglomerated- spherical	Antimicrobial activity against fungi and bacteria, antioxidant activity, non-linear optical activity	[65]
*Avicennia marina* (Mangrove)	25–35 nm	irregular, rough, and granular surface texture	Methylene Blue Removal, nitrate, and chemical oxygen Demand (COD) reduction	[66]
*Cissus rotundifolia*	1–100 nm	Spherical	Anticarcinogenic activity against *Streptococcus mutans* and *Lactobacillus *sp*.*	[67]
*Caesalpinia pulcherrima*	20–30 nm	Spherical	Photocatalytic dye degradation of methylene blue, antimicrobial activity against bacteria	[68]
*Mucuna pruriens*	5 μm to 500 nm	Triangular aggregation pattern	Antioxidant activity (DPPH, FRAP assays)	[69]
*Ocimum tenuiflorum* (Holy basil)	10.2–15.2 nm	knitted ball-like crystalline structure.	Enhancement of growth, physiology, enzymatic activities, essential oil content and yield	[70]
*Syzygium cumini* (Java plum)	12–30	Round and polydisperse	Nematicidal activity against root-knot nematode (*Meloidogyne incognita*), impact on plant growth and physiology	[71]
*Morus alba* (Mulberry)	28.34 nm	Anatase crystalline phase	Antimicrobial activity, UV protection, colorfastness to washing/rubbing/light	[72]
*Juniperus phoenicea*	10–30 nm	Spherical	Insecticidal activity against *Sitophilus oryzae*, antimicrobial activity, impact on the nutritional value of grains	[73]
*Terminalia bellirica*	420 nm	Spherical	Antioxidant activity, antimicrobial activity	[74]
*Psidium guajava* (Guava)	5–10 nm	Spherical	Antimicrobial activity against *E. coli* and *S. aureus*	[75]
*Salacia reticulata*	32–46 nm	flake-like structures	Antidiabetic, anti-inflammatory, antibacterial activities, developmental toxicity in zebrafish embryos	[76]
*Psidium guajava* (Guava)	10–30	irregular	Antimicrobial activity, anticancer activity against MG-63 cells, structural and optical characterization	[77]
*Commelina benghalensis*	150–200 nm	Roughly spherical	Photodegradation of methylene blue dye and sulfisoxazole antibiotic	[78]
*Moringa oleifera* (Moringa)	10 nm	Anatase form	Impact on germination and growth of spinach seeds	[79]
*Ipomoea carnea* (Morning glory)	7–10 nm.	spherical	Effect on growth, yield, and quality of black carrot	[80]
*Tinospora cordifolia*	18–20 nm	Spheroidal	Photocatalytic degradation of methylene blue dye,	[81]
*Tulbhagia violacea*	31–42 nm	Rectangular	Antioxidant activity, anticancer activity against HEK 293 and HeLa cell lines,	[82]
*Trianthema portulacastrum* *Chenopodium quinoa*	30–50 nm 40–60 nm	spherical round	Antifungal activity against *Ustilago tritici* (wheat fungal disease),	[83]
Mango leaf extract	10–30	Spherical (TN-2, TN-3), agglomerates (TN-1)	Antifungal activity against *Penicillium steckii*	[84]
*Terminalia catappa*	75.24 ± 1.12 nm	Monoclinic-shaped, clustered	Plant modulating ability on *Abelmoschus esculentus*, antioxidant activity, impact on plant growth and physiology	[85]
*Juniperus phoenicea*	10–30 nm	Spherical	Antimicrobial activity against various bacterial and fungal strains, anticancer activity against human ovarian adenocarcinoma cells	[86]
*Moringa oleifera* leaf	25–110 nm	spherical	Salinity Stress mitigation in wheat crops	[87]

#### Microbial synthesis

The green synthesis of NPs follows a bottom-up strategy, where NPs form through the oxidation/reduction of metallic ions facilitated by biomolecules secreted by microorganism entities. These biomolecules, including enzymes, proteins, sugars, carbohydrates, and others, play a crucial role in driving the redox processes that lead to the generation of nanoparticles [88]. Despite extensive research, a comprehensive understanding of the nanoparticle synthesis mechanism driven by microorganisms remains elusive as each type of microorganism interacts with metallic ions through various pathways [89]. The biochemical processes and interaction dynamics of a specific microorganism, coupled with environmental factors such as temperature and pH, ultimately influence the geometric parameters and form of the resultant nanoparticles [90].

The formation of nanoparticles can occur through either intracellular or extracellular mechanisms, contingent upon the specific type of microorganism involved [91]. Researchers have leveraged the potential of living cell extracts to pursue biological nanoparticle synthesis. The subsequent sections will concisely explore the primary biological pathways employed for this synthesis.

##### Bacterial-mediated TiO_2_-NPs biosynthesis

Bacteria are favored for nanoparticle synthesis due to their relatively undemanding conditions, uncomplicated purification processes, and prolific generation. Consequently, these microorganisms have emerged as extensively studied " nanomaterial production hubs“ [36]. Depending on their specific characteristics, various bacterial species can synthesize these NPs intracellularly or extracellularly (Table 3). The bacterial synthesis of TiO_2_-NPs is aided by their natural defense mechanisms, allowing them to adapt to environmental changes and resist certain metals. This process can occur through three primary methods: using whole cells, supernatants, or extracts. Supernatants, obtained after centrifuging bacterial cultures, contain various bioactive compounds such as enzymes, proteins, amino acids, polysaccharides, and carbohydrates, which are utilized as biocatalysts for NPs production [92]. This bacterial-mediated approach offers a versatile and efficient method, capitalizing on these microorganisms’ inherent properties and adaptability.

The biosynthesis of TiO_2_-NPs from Ti^3+^ ions (titanium precursors) by bacteria occurs through a three-step process: trapping, bioreduction, and capping. Initially, bacteria in the aqueous solution or surrounding medium trap Ti^3+^ ions. Subsequently, enzymes and proteins facilitate the reduction of these trapped ions into TiO_2_-NPs. Research has shown that microbial proteins containing functional groups such as –NH_2_, –SH, –COOH, and –OH plays a crucial role in stabilizing the synthesized TiO_2_-NPs [93]. These groups provide binding sites for Ti^3+^ ions and act as capping or stabilizing agents. The reduction of Ti^3+^ ions to NPs occurs either on the cell wall or within the bacterial intramembrane space. This process involves electron transfer from reduced compounds to inorganic compounds, promoting bacterial bioreduction of NPs. Finally, various bacterial biomolecules cap the reduced TiO_2_-NPs as natural stabilizing agents. This capping step is essential for maintaining NP stability, a critical factor in their synthesis and application [94].

Ti^3+^ (titanium salts) → Bacteria-Ti^3+^ ( Trapping).

Bacteria-Ti^3+^ + e^−^→ TiO_2_​-NP_s_ (Bioreduction)

TiO_2​_-NPs + microbial functional groups (–NH_2_, –SH, –COOH, –OH) → Capped TiO_2_​-NPs (Capping).

Among bacterial metabolites, exopolysaccharides (EPSs), which are heterogeneous organic biopolymers [95, 96] secreted into the extracellular environment [97] and have a vital function in providing protective microenvironments for bacteria [98, 99], enhancing their tolerance towards biotic and abiotic stresses while facilitating bacterial colonization [100, 101]. In biogenic nanoparticles, EPSs exhibit intriguing characteristics. One notable property is mucoadhesion, which facilitates neutral coating with low surface energy and prevents the recognition of non-specific protein receptors. Moreover, EPSs can adsorb metal cations onto their surface, a trait that aids in stabilizing and biosynthesizing metal nanoparticles. Their robust reducing and stabilizing capabilities make EPSs an attractive alternative biogenic substrate for synthesizing nanoparticles [102]. Upon interaction between metal ions and EPSs containing reducing sugars, the metal ions undergo chelation, followed by their reduction and stabilization facilitated by various functional moieties. Prominently, polyanionic groups, chiefly -COOH and -OPO_3_^2−,^ are well-documented to partake in reducing and stabilizing metal nanoparticles. Furthermore, electrostatic interactions between metal cationic species and anionic EPS groups like -COOH and -OPO_3_^2−^ have been cited as advantageous for nanoparticle synthesis. Among these functional entities, -OH, -COOH, -OPO_3_^2−^, hemiacetal, and amino terminals have been proposed to reduce metal ions from precursor salts, yielding the corresponding nanoparticles. Notably, -OH groups coordinate with metal ions, whereas their oxidation to form carbonyl and -COOH groups is pivotal during the reduction process and nanoparticle formation [103]. Moreover, the abundance of -OH and hemiacetal end groups in EPSs facilitates the reduction and stabilization of nanoparticles, rendering them more suitable for diverse applications. Additionally, monosaccharides like glucose, galactose, mannose, and fructose have been mentioned to reduce metal ions, contributing to the mechanisms involved in nanoparticle synthesis observed with other EPSs [104].

Also, Actinomycetes have the ability to produce a diverse array of secondary metabolites, have emerged as promising sources for the biosynthesis of nanoparticles with desirable surface characteristics and size control [105]. These microorganisms possess the remarkable capability to facilitate the production of metallic and metal oxide NPs through either intracellular or extracellular methodologies [106] (Table 3). The extracellular approach has garnered significant commercial interest due to its advantage of minimizing polydispersity, a crucial factor in nanoparticle synthesis [107]. Notably, actinomycetes are a class of high-content guanine and cytosine microorganisms primarily exploited for their antibiotic production capabilities [108]. However, their versatility extends beyond antibiotics, as several researchers have successfully synthesized TiO_2_-NPs by harnessing the metabolic pathways of these remarkable microorganisms [109]. This further highlights the potential of actinomycetes as a valuable resource for the green synthesis of nanoparticles with diverse applications.

Among the recent examples, one study reported the synthesis of spherical-shaped titanium TiO_2_-NPs, ranging in size from 30 to 70 nm, using *Streptomyces* sp. HCl. The developed TiO_2_-NPs were tested as antimicrobials against various pathogenic microorganisms, including *Staphylococcus aureus*, *Escherichia coli*, *Candida albicans*, and *Aspergillus niger*. The researchers concluded that the synthesized TiO_2_-NPs exhibited higher antimicrobial properties against bacterial species compared to fungal species [110]. Another study reported the synthesis of TiO_2_-NPs using the marine actinobacteria *Streptomyces bluensis* as the biological source. The precursor for the nanoparticle synthesis was Ti(OH)_4_. The resulting spherical TiO_2_-NPs exhibited an average size of 37.54 nm and exhibited high potential to degrade azo dyes, including AR-79 and AR-80, with percentages of 84% and 85%, respectively [111]. Table 3 shows some bacterial and actinomycetes species that were utilized for TiO_2_-NP synthesis.

**Table 3:** Bacterial and actinomycetes species-mediated biosynthesis of TiO_2_-NPs showing promising biomedical and biotechnological applications

Source	Size	Shape	Applications/Properties Studied	Reference
**TiO**_**2**_ **-NPs fabricated by bacterial strains**				
*Staphylococcus aureus* (G + ve)	~ 20 nm (average diameter)	Smooth and spherical	Antibacterial and antibiofilm against *Bacillus subtilis* and *Escherichia coli*	[112]
*Lactobacillus rhamnosus* (G + ve)	3–7 nm	Spherical	Antifungal against isolated fungal strains, Biocompatible towards WI38 and HFB4 cell lines	[113]
*Bacillus subtilis* MTCC 8322 (G + ve)	80–120 nm	Spherical to irregular	Photocatalytic dye degradation (Methylene Blue and Orange G)	[114]
*Bacillus subtilis* (G + ve)	70.17 nm	spherical	Improved mechanical properties of Glass Ionomer Cement (GIC) for dental applications	[115]
*Paenibacillus *sp*.* HD1PAH and *Cyperus brevifolius.*	17.11 nm 29.39 nm	Spherical Granular	Anthracene biodegradation, soil enzyme activities increased	[116]
*Halomonas elongata* IBRC-M 10,214 (G-ve)	104.63 ± 27.75 nm	Spherical	Antibacterial against *E. coli* and *S. aureus*	[117]
*Lactobacillus johnsonii* (G +ve)	4–9 nm	Irregular	Desalinization and surface cleaning	[118]
*Bacillus amyloliquefaciens* (G +ve)	22.11–97.28 nm	Spherical	Photocatalytic dye degradation (Reactive Red 31), enhanced by doping	[119]
*Aeromonas hydrophila* (G-ve)	40.50 nm	Smooth, spherical, uneven	Antibacterial against *S. aureus* and *S. pyogenes*	[120]
*Propionibacterium jensenii* (KC545833) (G +ve)	< 80 nm	uniform size and anatase form	Collagen stabilization for wound dressing	[121]
*Bacillus subtilis* (FJ460362) (G +ve)	10–30 nm	Mostly spherical	Photocatalytic control of aquatic biofilm	[122]
**TiO**_**2**_ **-NPs formed by actinomycetes strains**				
*Streptomyces* sp. HC1	30–70 nm	Spherical	Antimicrobial activity, antibiofilm activity	[110]
*Streptomyces bluensis*	58.3 nm	spherical	Azo dye degradation	[123]
*Saccharopolyspora spinosa*	23.3 nm	Spherical	Antimicrobial	[124]

##### Fungal-mediated TiO_2_-NPs biosynthesis

**Fig. 2:**
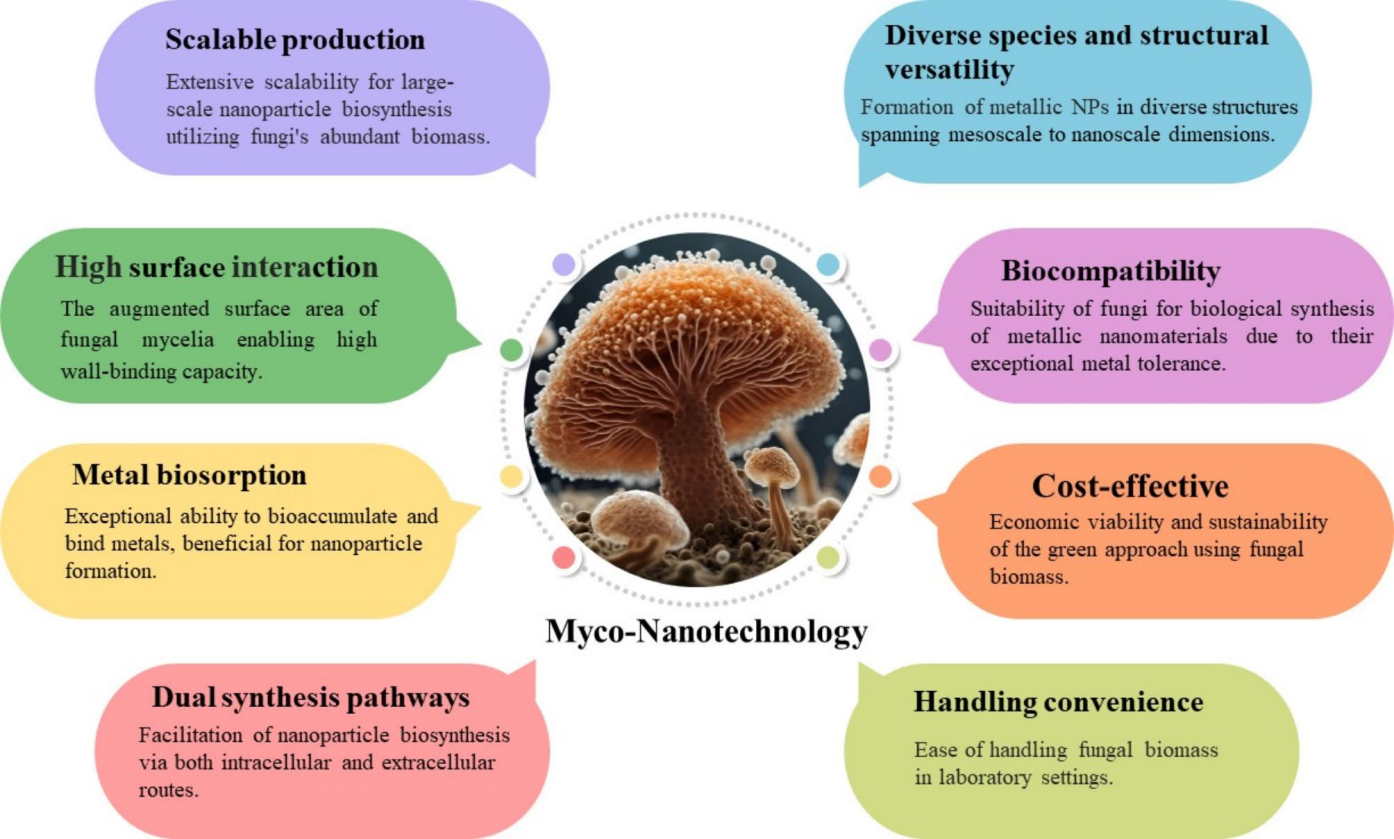
Promising characteristics of fungi as a green tool for NPs synthesis

Fungi have gained widespread nanoparticle biosynthesis adoption due to their metabolites’ remarkable efficacy in fabricating diverse nanoparticles. They represent a valuable addition to the repertoire of microorganisms employed for nanoparticle production. The extensive utilization of various fungal species can be attributed to their capacity to secrete substantial quantities of proteins or enzymes, coupled with their ease of handling in laboratory settings [125]. The utilization of fungi for synthesizing metallic nanoparticles has garnered substantial attention due to their possessing certain advantageous traits surpassing other organisms. The ability to readily scale up and streamline downstream processes, the economic viability, and the presence of mycelia with an augmented surface area constitute valuable advantages that warrant due consideration [126]. Furthermore, fungi have gained heightened interest owing to their involvement in the biological synthesis of metallic nanomaterials, facilitated by their exceptional tolerance and remarkable ability to bioaccumulate metals [127] (Fig. 2).

The synthesis of nanoparticles employing fungi and their biotechnological applications, particularly in medicine, are encompassed within the realm of myco-nanotechnology. This scientific term, representing the convergence of “mycology” and “nanotechnology,” holds significant potential due to the vast diversity and range of fungal species [128]. Fungi can form nanoparticles in diverse structures spanning mesoscale to nanoscale dimensions via enzymatic reduction, either extracellularly or intracellularly, and through biomimetic mineralization processes. Their extensive scalability has led to a distinct preference for their utilization in nanoparticle synthesis, exemplified by techniques such as thin solid substrate fermentation. Owing to the remarkable secretory capabilities of fungi, producing abundant quantities of extracellular enzymes or proteins becomes viable [129]. Furthermore, the economic feasibility and sustainability of employing biomass present another advantage for the implementation of a green approach, enabled by fungal entities or byproducts, in the fabrication of metallic nanomaterials [130]. Here are some examples of different fungal species used for the biosynthesis of TiO_2_-NPs, referring to their activities (Table 4).

Yeasts, a class of fungi belonging to the ascomycetes group, have demonstrated remarkable potential for the synthesis of nanoparticles. Biological processes possess a superior ability to control the morphology of materials. During the logarithmic growth phase, the yeast *Schizosaccharomyces pombe* facilitated the synthesis of semiconductor nanocrystals [126]. Nanoparticles synthesized extracellularly by yeasts offer a wide range of advantages, as the protein-mediated interactions between the yeasts and nanoparticles facilitate subsequent downstream reactions and modifications [131]. The entire yeast family exhibits the remarkable capability of aggregating various heavy metals. They can sequester substantial amounts of toxic metal ions. Numerous studies have concluded that the mechanisms employed by these species to counteract the toxic effects of heavy metals involve the extracellular production of polysaccharides or peptides. These biomolecules play a crucial role in either managing the cell’s permeability barrier against heavy metals or actively effluxing them from the cell [132]. For direct intracellular delivery of metal ions, yeast cells need to be disrupted to avoid negative or lethal consequences. The toxicity to cells can be mitigated by either maximizing the retention of metal ions within the cells or by exposing them to metals that do not possess the same level of toxicity as lead, mercury, and cadmium ions [133].

The significant variations observed in size, particle distribution, monodispersity, and characteristics of the synthesized nanoparticles can be attributed to the diverse mechanisms employed by different yeast strains from various families for nanoparticle formation. In yeast cells, a detoxification mechanism involving glutathione (GSH) and two classes of metal-binding proteins, namely metallothioneins and phytochelatins (PC), is utilized to facilitate the formation of nanoparticles [89]. In most yeast species, these molecules (GSH, metallothioneins, and PC) facilitated the formation pathway of nanoparticles and stabilized the resulting compounds. The resistance mechanism was defined as the ability of yeast cells to convert the absorbed metal ions into non-toxic polymer complexes. The nanoparticles formed by yeasts are commonly referred to as “semiconductor nanocrystals” or “quantum semiconductor nanocrystals”. Recent research has revealed that yeasts can also synthesize various other types of nanoparticles [126]. More examples of biosynthesis of TiO_2_-NPs using yeast strains were summarized in (Table 4).

**Table 4:** Examples of biosynthesis of TiO_2_-NPs using different fungal species with reference to their size, shape, and activities

Species	Size	Shape	Applications / Properties Studied	Reference
**TiO**_**2**_ **-NPs produced by multicellular fungi**				
*Aspergillus eucalypticola* SLF1	33 nm	Mesoporous, anatase phase	Photocatalytic, antimicrobial, antioxidant activity	[134]
*Paraconiothyrium brasiliense*	57.39 ± 13.65 nm	Spherical	Antibacterial, antibiofilm properties	[135]
*Fomitopsis pinicola*	80–120 nm	Irregular with a rough surface	Antibacterial and anticancer activity	[136]
*Trichoderma viride*	10.4 to 45.8 nm (size range), 26.619 ± 7.577 nm (mean size)	Elliptical to spherical	Larvicidal, antifeedant, pupicidal activity against *Helicoverpa armigera*	[137]
*Trichoderma citrinoviride*	10–400 nm	Irregular, triangular, pentagonal, spherical, rod-shaped	Antibacterial activity against *Pseudomonas aeruginosa*, antioxidant potential	[138]
*Trichoderma harzianum*	50.0 nm	Spherical	Enhanced growth of *T. harzianum*, inhibitory against *Sclerotinia sclerotiorum*	[139]
*Aspergillus niger* DS22	10.4 to 45.8 nm, 26.619 ± 7.577 nm (mean size)	Elliptical to spherical	Antibacterial, anti-inflammatory, wound-healing activities	[140]
*Aspergillus flavus*	62–74 nm	Spherical, oval	Antibacterial activity	[141]
*Metarhizium anisopliae*	9.50 nm	Spherical	Larvicidal, pupicidal, antifeedant activity against *Spodoptera frugiperda*	[142]
*Hypsizygus ulmarius*	~ 80 nm	Spherical	Antibacterial and anticancer potential	[143]
*Pleurotus djamor*	31 nm	Spherical	Mosquito larvicidal, antibacterial, anticancer effects	[144]
*Streptomyces* sp. HC1	30 to 70 nm	Spherical	Antimicrobial and antibiofilm activity	[110]
*Alternaria solani*	15 nm	Agglomerated	Antimicrobial, anticoagulant, antiplatelet, hemolytic and cytotoxicity properties	[145]
**TiO**_**2**_ **-NPs produced by yeasts**				
*Saccharomyces cerevisiae* (Baker’s yeast)	8–35 nm	Individual aggregate	Photocatalytic antimicrobial activity	[146]
*Saccharomyces cerevisiae*	6.7 ± 2.2 nm	Spherical	Photocatalytic activity, antimicrobial activity	[147]

##### Viruses-mediated TiO_2_-NPs biosynthesis

Viruses provide an important illustration of how nanosized particles can be synthesized. So far, viruses have produced nanotubes and nanorods [148]. Research studies and experiments have demonstrated that plant viruses and certain bacteriophages can be readily isolated and subjected to further processing [149]. However, not all virus components can synthesize nanoparticles, and the underlying reasons for this need to be thoroughly investigated and understood [150]. One study reported the successful formation of chalcogenide nanocrystals within genetically modified virus-like particles [151]. Leveraging the self-assembly capabilities of the genetically modifiable M13 virus, TiO_2_ nanostructures with controlled sizes between 20 and 40 nm were synthesized under ambient conditions, enabling their homogeneous distribution and percolated networks suitable for exploiting their promising photo-electrochemical properties [152] (Table 5).

##### Algae-mediated fabrication of TiO_2_-NPs

Algae are capable of accumulating significant quantities of heavy metals, an attribute that enables them to be utilized for the biosynthesis of metallic and metal oxide NPs [153]. Algae have found widespread application in the synthesis of TiO_2_-NPs due to their ready availability and effectiveness. Beyond just enzymes and proteins, these phytosynthetic organisms also possess carotenoids and various pigments involved in photosynthesis, which contribute significantly to the physio-assisted (algae-mediated) production of TiO_2_-NPs. However, algal-based synthesis methods for these nanoparticles are not as well-established compared to bacterial synthesis routes [154]. Additionally, algae can synthesize nanoparticles utilizing their extracts or supernatants, which are rich in secondary metabolites. These extracts obviate the need for live algal cultures in nanoparticle synthesis [155].

In one study, TiO_2_-NPs were synthesized using the algae *Spirulina platensis* [156]. Moreover, another study reported the synthesis of spherical-shaped TiO_2_-NPs ranging from 90 to 150 nm in size by utilizing an extract from *S. platensis* [157]. Also, TiO_2_-NPs were synthesized from the seaweed *Sargassum wightii*, and their efficacy was evaluated for killing the larvae of vectors that transmit malaria and filariasis [158]. Another investigation involved the synthesis of negatively charged TiO_2_-NPs with cubic, square, and spherical shapes ranging approximately 50–90 nanometers in size, utilizing *Sargassum myriocystum* as the source material. Additionally, the researchers in this study assessed the antimicrobial properties of the synthesized titanium dioxide nanoparticles [159]. Table 5 summarizes some examples of the biosynthesis of TiO_2_-NPs using different algal strains.

**Table 5:** Examples of the biosynthesis of TiO_2_-NPs using viruses and algae with noted size, shape, and applications

Source	Size		Shape	Activity	Reference
**Viruses**					
M13 bacteriophage		20–40 nm	quasi-spherical	photo-electrochemical properties	[152]
**Algae**					
*Bostrychia tenella* (Red macroalga)		22.86 nm	Uniform, monodispersed	Antifouling activity	[160]
*Carteriospongia foliascens* (Marine sponge)		8.3 nm	uniform, monodispersed particles	Antifouling activity	
*Sargassum wightii* (Brown macroalga)		20–80	spherical	Larvicidal activity against mosquito malaria’s vector	[161]
*Phaeodactylum tricornutum* (Microalga)		50 nm	spherical	Antimicrobial activity, antistatic properties, cytotoxicity against cancer cell lines	[162]
*Sargassum myriocystum* (Brown macroalga)		50–90 nm	Cubic, square, spherical	larvicidal activity against mosquito larvae, photocatalytic dye degradation	[163]
*Spirulina platensis* (Microalga)		4.62 nm	Spherical, dispersed irregularly	Antifungal Activity	[164]
*Spirulina platensis*		90–150 nm	spherical	Antimicrobial activity	[157]

## Factors modulating biosynthesis

The shaping and resizing of metallic and their oxide nanomaterials seem to be influenced by their environmental conditions or altered by the presence of functional molecules [165]. Researchers have explored modifying various synthesis parameters, such as thermal conditions, acidity/alkalinity, incubation duration, oxygenation, ionic strength, redox state, mixing proportions, and irradiation, to optimize the production of nanoparticles. Both chemical and physical factors govern the dimensions and morphology of nanoparticles. Achieving optimal conditions for metal ion concentration, thermal environment, and acidity/alkalinity is crucial during the synthesis process [166]. The properties of nanoparticles formed via biological methods are significantly influenced by the incubation time of the reaction medium. Prolonged incubation can lead to variations in characteristics, potentially due to particle aggregation or shrinkage [46]. Consequently, the particles’ self-life or stability may impact their functional potential over extended periods. Therefore, in addition to the synthesis parameters like thermal conditions, acidity/alkalinity, substrate concentration, and exposure duration to the substrate, the incubation time is a crucial factor that governs the characteristics and potential of biologically synthesized nanoparticles [167]. Adjusting these synthesis parameters can modulate the rate of nanoparticle formation within cells and their eventual size to some extent. In the subsequent subsections, precursor concentration, temperature, and pH will be further explained, since these key parameters contribute to the optimization and the efficiency of the biogenic process as they significantly influence the nucleation rate, reaction kinetics, aggregation, and stabilization of the desired nanoparticle characteristics.

### Effect of Precursor and reducing Agent concentrations on nanoparticle size and agglomeration

Precursor and reducing agent concentrations crucially determine the synthesized nanoparticles’ size [168]. Initially, excessive reducing agents bind to preformed nuclei, amplifying secondary ion reduction on their surfaces. This accelerates nanoparticle growth, yielding larger sizes at higher reactant concentrations [169]. However, an overly high reducing agent concentration adversely promotes nanoparticle bridging and aggregation. This arises when an abundance of metal ions adsorbs onto nuclei surfaces, facilitating uncontrolled secondary reduction and agglomerated growth [170]. Therefore, striking the right balance in reactant concentrations is vital to avoid aggregation issues and achieve monodisperse nanoparticles with the desired size [171].

### Temperature effects on nanoparticle size and dispersity

Temperature plays a crucial role in determining nanoparticle size during synthesis. Higher temperatures tend to promote nucleation, leading to the formation of numerous smaller nanoparticles. In contrast, lower temperatures favor slower growth processes, resulting in fewer but larger nanoparticles [172]. However, increasing the overall reaction temperature accelerates the total reaction rate [173]. Notably, temperature exhibits contrasting effects on the size under sufficient versus insufficient precursor conditions due to differing influences on nucleation and growth kinetic constants [174]. As temperature increases, the enhanced reduction rate consumes most metal ions in nuclei formation, hindering secondary reduction on preformed nuclei surfaces. Consequently, elevated temperatures yield smaller, highly dispersed nanoparticles with improved yield [175].

### Influence of pH on nanoparticle size, shape, and Colloidal Stability

The pH during nanoparticle synthesis exerts a profound influence not only on the size but also on the shape of the resulting particles [176]. The pH governs the local surface characteristics of nanoparticles [177] by facilitating the protonation and deprotonation of molecular atoms during the nucleation and growth stages [178]. Notably, nanoparticles synthesized under lower pH conditions exhibit less regularity in shape and a tendency toward aggregation. However, by tailoring the pH conditions during synthesis, nanoparticles with the desired size and uniform shape can be produced [179]. Conversely, in the alkaline pH range, nanoparticles form a well-dispersed cluster distribution in the colloidal stage, thereby preventing aggregation [180].

## Characterization of nanoparticles

The physicochemical characterization of synthesized nanoparticles is a crucial step that demands meticulous attention before their practical applications. Investigating properties such as size, shape, surface area, homogeneity, and stability provides valuable insights into these nanoscale systems, enabling better control over nanoparticle synthesis for commercial purposes [181]. Various characterization techniques are employed, each serving a distinct function [182]. The color change test offers a simple visual indication of nanoparticle formation. UV-visible spectrometry analyzes the optical properties and confirms the presence of nanoparticles. In the case of TiO_2_-NPs synthesized by bacteria, they exhibit UV-Vis absorption peaks in the range of 300–400 nm. Most of these peaks are observed between 350 and 400 nm, whereas in some cases, the peaks are found below 350 nm. This variation in peak position is attributed to the presence of different biomolecules involved in the synthesis process and the size and shape of the NPs, which affect their surface plasmon resonance. Fourier transform infrared spectroscopy (FT-IR) elucidates the chemical composition and identifies functional groups that facilitate the stabilization and surface modification/capping of the synthesized TiO_2_-NPs.

Electron microscopy techniques, including transmission electron microscopy (TEM), high-resolution TEM (HR-TEM), scanning electron microscopy (SEM), and field emission-SEM (FE-SEM), provide detailed visualization and structural analysis of nanoparticles. Also, they provide insights into the association of carbon-based biomolecules with the synthesized NPs [183]. In SEM, the non-metallic regions appear darker due to electron deficiency, while in TEM, these areas appear brighter compared to the darker titanium element. Additionally, the elemental analyzer integrated with electron microscopy facilitates the determination of the chemical composition and purity assessment of the synthesized TiO_2_-NPs. Complemented by data from (FTIR) and (XRD), electron microscopy can reveal the presence and nature of biomolecules associated with the TiO_2_-NPs [184].

Energy-dispersive X-ray spectroscopy (EDX) mapping reveals elemental composition and distribution within nanoparticles. Dynamic light scattering (DLS) measures the size distribution and aggregation behavior of nanoparticles in solution [185].

Powder X-ray diffraction (XRD) is crucial for characterizing the synthesized TiO_2_-NPs as it determines their crystalline structure and phase composition. TiO_2_-NPs can exist in three different phases: anatase, rutile, and brookite. The anatase phase exhibits a sharp peak near two theta values (2θ) of 25–26°, while the rutile phase is identified by a peak around 2θ value of 27–28°. By analyzing the specific peak positions and intensities in the XRD pattern, the presence of these phases in the synthesized TiO_2_-NPs can be determined. Vibrating sample magnetometry (VSM) evaluates their magnetic properties. Thermogravimetric analysis (TGA) assesses thermal stability and quantifies components within nanoparticle samples. Zeta potential reflects the surface charge of NPs, influencing their suspension stability. Higher absolute values imply enhanced stability through electrostatic repulsion. Contact angle measurements determine the extent to which a liquid spreads on a solid surface, providing insights into the surface energy and hydrophobicity of nanoparticles [186]. Moreover, the 2D shape and 3D shape were detected and investigated by atomic force microscopy (AFM), whereas the chemistry of TiO_2_-NPs surface, state of chemical and electronics within NPs, and elemental composition were assessed by X-ray photoelectron spectroscopy(XPS) [187].

These techniques collectively offer comprehensive characterization, enabling researchers to thoroughly understand the synthesized nanoparticles and optimize their synthesis for diverse applications.

## Biomedical and biotechnological applications of TiO_2_-NPs

**Fig. 3:**
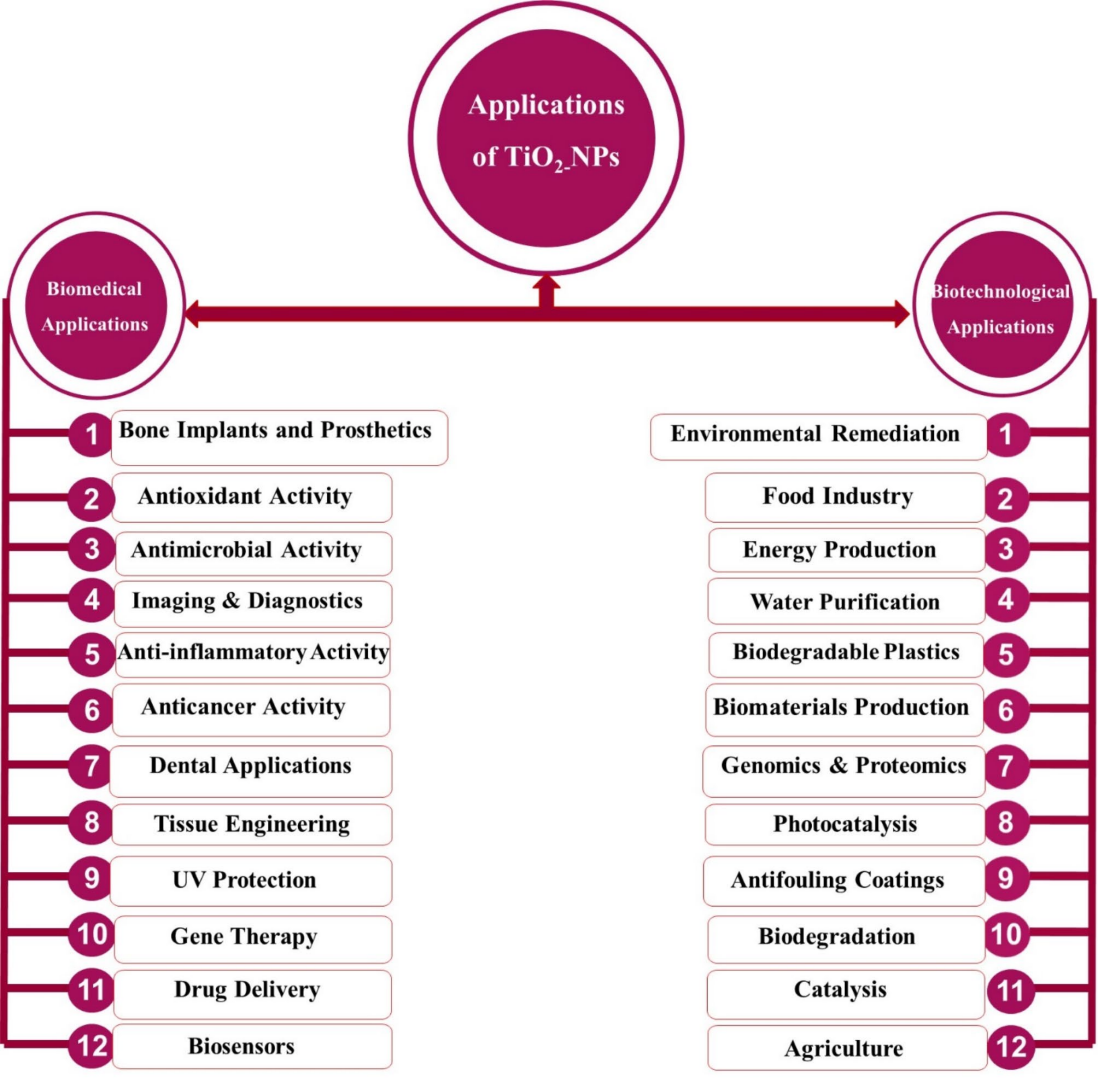
Flowchart enumerates the biomedical and biotechnological applications of TiO_2_-NPs

Within the rapidly evolving landscape of nanotechnology, the realm of biomedical applications has witnessed remarkable progress fueled by extensive research endeavors. Nanoparticles have garnered significant attention due to their distinctive physicochemical properties, which render them advantageous across a diverse array of fields, including pharmaceutical formulations, diagnostic tools, personal care products, and electronic devices [188]. The unique characteristics inherent to nanomaterials, particularly their minuscule size ranging from 1 to 100 nm, heightened reactivity, and immense surface area, have unlocked novel avenues for exploration and innovation [189]. These properties facilitate their cellular entry and interaction with biomolecular and cellular pathways, rendering nanomaterials invaluable assets in the realm of drug therapeutics [190](Fig. 3).

TiO_2_ emerges as a highly versatile material, rendering it an attractive choice across a wide array of applications due to its remarkable mechanical and photochemical characteristics [191]. In the realm of cosmetics, TiO_2_ finds utility in formulating anti-aging and skin-brightening products, harnessing its unique characteristics [192]. Similarly, its optoelectronic properties make it a valuable component in the fabrication of devices and sensors within the electronics industry [193]. Furthermore, the medical field has embraced TiO_2_ for its potential in targeted drug delivery systems, antimicrobial activity, treatment of cancer cells, biosensors, dental treatment, anti-inflammatory activity, etc. (Fig. 3). The following subsections comprehensively explore some biomedical and biotechnological applications of TiO_2_-NPs, highlighting their remarkable versatility and vast potential across diverse domains.

### Antioxidant activity

TiO_2_-NPs, especially those formed by plant extract that impaired various functional groups, showed high antioxidant activity to be utilized for decreasing the deleterious effects of free radical ions [194]. These TiO_2_-NPs were able to scavenge free radicals, such as 2,2′-casino-bis (3-ethylbenzothiazoline-6-sulfonic acid) (ABTS) and 2,2-Diphenyl-1-(2,4,6-trinitrophenol) hydrazine-1-yl (DPPH), within a reduced timeframe, demonstrating their antioxidant capabilities (Table 6). TiO_2_-NPs are also reported to exhibit protective effects against reactive oxygen species (ROS). Plant-mediated synthesis methods result in TiO_2_-NPs with functional groups such as phenols and tannins, contributing to their stabilization and enhanced antioxidant potential. These functional groups present on the surface of the nanoparticles play a crucial role in scavenging free radicals and mitigating oxidative stress [195].

In a study, the green synthesis of TiO_2_-NPs using *Psidium guajava* (guava) leaf extract exhibited superior antioxidant activity compared to ascorbic acid. This enhanced antioxidant potential was attributed to the presence of phenolic compounds in the aqueous leaf extract (85.4 mg/g) and the synthesized TiO_2_-NPs (18.3 mg/g).

The phenolic content in the plant extract played a crucial role in the formation and stabilization of the nanoparticles, contributing to their remarkable antioxidant properties [196]. Similarly, *Artemisia haussknechtii* leaves synthesized TiO_2_-NPs with strong antioxidant properties. The synthesized NPs have been tested through various assays, indicating a remarkable DPPH scavenging activity of 68.43% at a concentration of 500 µg/ml. The reducing power of TiO_2_-NPs was greater than ascorbic acid (control), as determined by the reducing power assay to donate an electron [197]. In another study, TiO_2_ nanoparticles, produced using fruit peel agro-waste, possess antioxidant potential. The study utilized various scavenging assays such as DPPH free radical, H_2_O_2_ free radical, and NO, as well as the reducing power assay. The results showed that TiO_2_ nanoparticles exhibited dose-dependent antioxidant effects compared to ascorbic acid, which was used as a control [198].

**Table 6:** Antioxidant activity of green synthesized TiO_2_-NPs

TiO_2_-NPs Synthesized by	Antioxidant Activity/ Assay used	Reference
*Psidium guajava* (guava) leaf extract	Superior to ascorbic acid/(TiO_2_-NPs:18.3 mg/g)	[196]
*Artemisia haussknechtii*	DPPH scavenging activity: (68.43% -500 µg/ml of TiO_2_-NPs)	[197]
fruit peel agro-waste	Dose-dependent antioxidant effects (DPPH, H_2_O_2_ free radical, NO, and reducing power assays)	[198]
*Lawsonia inermis* Leaf extract	5-100 mg/ml of TiO_2_-NPs enhanced DPPH scavenging and 82% reduction in hydrogen-mediated hemolysis	[199]
*Syringodium isoetifolium*	Strong antioxidant activity in DPPH and ABTS assays	[200]
*Tinospora Cordifolia*	90% DPPH scavenging assay	[201]
*Trichoderma citrinoviridae*	50–100 µg/ml of tested TiO_2_-NPs in DPPH were potent than standard gallic acid	[138]
*Coleus aromaticus*	100 µg/ml of TiO_2_-Nps tested caused 89% scavenging in DPPH assay	[202]
*Withania somnifera* *Eclipta prostrata*	DPPH scavenging activity (68.43% at 500 µg/ml)	[203]
*Laurus nobilis *(bay leaf)	DPPH 46.71% at 200 µg/ml and H_2_O_2_ of 58.45% at 50 µg/ml	[203]
*Terenna asiatica*	IC_50_ = 80.21 µg/µL in the DPPH assay	[204]
*Achillea wilhelmsii* C. Koch	ROS generation (215.4%) and decreased MMP (72%)	[205]
*Terminalia catappa* bark extract	47% reduction in MDA content	[85]
*Pithecellobium dulce* *Lagenaria siceraria* Leaves extracts	52% and 45% for DPPH inhibition respectively	[206]
*Malva parviflora* extract	85% DPPH inhibition and 90% scavenging for ABTS.	[207]
*Tulbhagia violacea* Leaf extracts	50 µg/mL of TiO_2_-NPs showed IC_50_ = 32.7 in DPPH assay	[82]
*Limonia acidissima* Peel extract	(ROS) release and (MMP) damage at 70 µg/ml of TiO_2_-NPs	[208]

**DPPH**:2,2-Diphenyl-1-picrylhydrazyl; **NO**: Nitric Oxide; **ABTS**: 2,2’-Azinobis-(3-ethylbenzothiazoline-6-sulfonic acid); **ROS**: reactive oxygen species; **MMP**: Mitochondrial Membrane Potential; **MDA**: Malondialdehyde

### Antimicrobial activity

#### Antibacterial activity

The indiscriminate use of antibiotics has resulted in the emergence of multidrug-resistant bacterial strains, which has become a significant cause for concern regarding food safety and human health [209]. In the quest for novel antibacterial agents, metal oxide nanoparticles have garnered significant interest from researchers, with biofilm formation being identified as a major factor contributing to antibiotic resistance [210]. Consequently, scientific investigations have shifted focus to explore the antimicrobial capabilities of nanoparticles composed of metals and metal oxides.

**Fig. 4:**
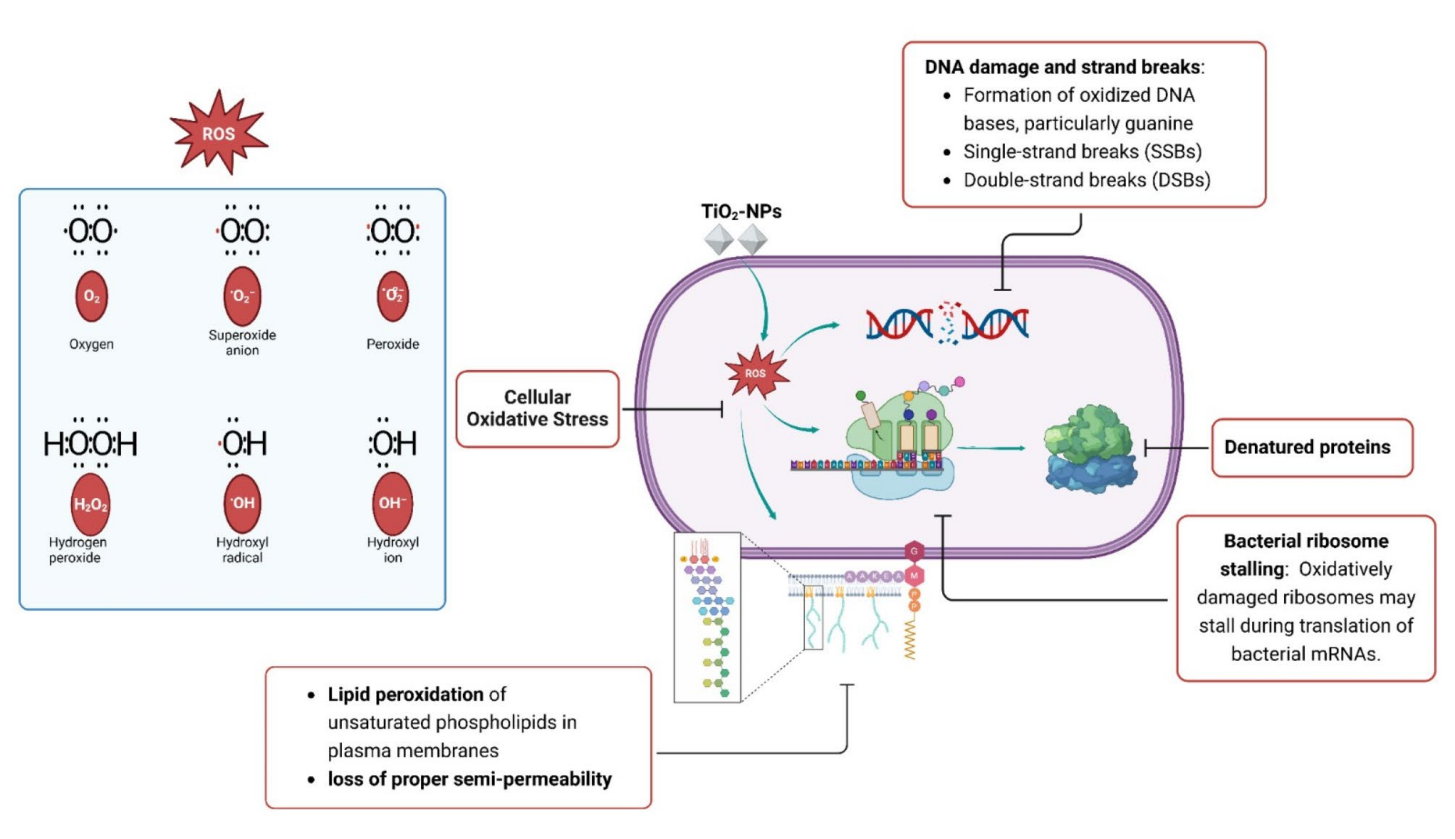
Various antibacterial mechanisms of biogenic TiO_2_-NPs

TiO_2_-NPs exhibit photocatalytic antimicrobial activity when exposed to UV irradiation with a wavelength below 385 nm. However, the effectiveness of this antimicrobial activity displayed by TiO_2_-NPs is contingent upon the thickness of the microbial cell surface [211]. TiO_2_-NPs exhibit antibacterial activity through the generation of reactive oxygen species (ROS), such as hydroxyl radicals (OH^-^), superoxide anions (O2^•-^), and hydrogen peroxide (H_2_O_2_) (Fig. 4). These ROS induce oxidative stress on the bacterial cell membrane, inducing lipid peroxidation in the plasma membrane’s unsaturated phospholipids. Consequently, the bacterial membrane sustains damage. Additionally, the photocatalytic activity of TiO_2_-NPs disrupts crucial biological processes within bacteria, including respiration, oxidative phosphorylation reactions, and the maintenance of semi-permeability [212]. Figure 4 presents various antibacterial mechanisms of TiO_2_-NPs used to inhibit bacterial growth.

A research investigation examined the impact of TiO_2_-NPs on biofilm formation by Methicillin-Resistant *Staphylococcus aureus* (MRSA) using a tissue culture plate method (Table 7). Among the 30 isolates evaluated, 22 demonstrated strong biofilm formation capabilities, while 2 exhibited weak biofilm formation. The study revealed that TiO_2_-NPs at a concentration of 500 µg/mL inhibited the growth of both strong and weak MRSA biofilm formers, suggesting the potential of TiO_2_-NPs as viable antibacterial options [213]. In another study, TiO_2_-NPs were employed in combination with antibiotics ceftazidime and cefotaxime against multidrug-resistant *Pseudomonas aeruginosa*. The bacterial samples were isolated from sputum, pus, and bronchoalveolar lavage. Upon 1-hour UV light exposure, a bactericidal effect was observed at TiO_2_-NPs concentrations exceeding 350 µg/ml. The minimum inhibitory concentrations of TiO_2_-NPs were six-fold higher compared to the antibiotics. Consequently, the combined application of antibiotics and TiO_2_-NPs synergistically augmented the antimicrobial activity [214].

Recently, the synthesis of TiO_2_-NPs-based nanocomposites is characterized by their high activity in the biomedical and food packaging sectors (Table 7). Their production encounters challenge due to the inherent dissimilarities between the inorganic and hydrophilic characteristics of the nanoparticles and the hydrophobic nature of polymer matrices. Nonetheless, antimicrobial nanocomposites derived from titania have garnered substantial interest in recent times [215]. A research endeavor delved into the electrochemical fabrication of silver-titania nanocomposites, striving to augment their photocatalytic characteristics while simultaneously amplifying their antifungal and antibacterial activities [216]. Another study focused on the development of paraffin and silver-coated titania nanoparticles (TiO_2_/AgNPs) embedded in a polyethylene nanocomposite for food packaging purposes. Nanocomposite films were fabricated by melt-blending 3% and 5% of TiO_2_/Ag NPs into low-density polyethylene. The findings revealed that the addition of 5% TiO_2_/Ag NPs resulted in a substantial decrease in bacterial growth [217]. Therefore, the incorporation of TiO_2_ nanoparticles, whether used independently or integrated with polymers, results in the suppression of microbial growth, thereby preventing food spoilage and enhancing the shelf-life of food products [218].

**Table 7:** Summarizing the antimicrobial activity including antibacterial, antifungal, and antiviral of synthesized TiO_2_-NPs

TiO_2_-NPs nature	Size	Shape	Target and finding	References
**Antibacterial**				
*Terminalia chebula*	56 nm	tetragonal	Decreased biofilm of *St. mutans*	[219]
*Rosa davurica*	146 ± 3 nm	irregular	Bacterial biofilm inhibition of *S. aureus* and *B. cereus*	[220]
*Cynodon dactylon*	< 100 nm	firmly agglomerated	Antibacterial effect against *A. baumannii* and *St. aureus*	[221]
*H. thelbiecea* *Ananos seneglensis*	40 nm 50 nm	spherical crystalline	Bacterial cell membrane damage	[222]
*Azadirachta indica Ficus benghalensis Syzygium aromaticum*	10–33 nm	tetragonal crystalline	Bactericidal effect against *Streptococcus mutans* and *Citrobacter freundii*	[223]
*Ocimum americanum* L. leaf	25 nm	spherical	Bactericidal action against *Clostridium perfringens*,* S. paratyphi* and *K. pneumoniae*	[224]
*Pleurotus djamor*	31 nm	spherical	Antibacterial effect against *Pseudomonas fluorescens* and *C. diphtheriae*	[144]
Commercial	< 50 nm	NA	Inhibited MRSA biofilm formation	[213]
microemulsion	9 nm	anatase structure	Bactericidal effect on *Pseudomonas aeruginosa*	[214]
Electrochemical (sacrificial anode)	TiO_2_ anatase (15.6 nm) Ag-TiO_2_ (15.6 nm)	spherical	Enhanced antifungal and antibacterial activities	[216]
sol-gel method	10–15 nm	spherical	Used in food packaging to prevent spoilage & decreased bacterial growth	[217]
**Antifungal**				
*Curcuma longa*	92.6 nm	anatase	Increased resistance to damping off fungal disease by *F*. *graminearum*	[225]
Commercial	70–130 nm	Anatase crystal	*C. albicans* was inhibited by 65%	[226]
Ball milling method	108–130 nm	irregular	hyphal lysis of *Macrophomina phaseolina*	[227]
*Bacopa monnieri*	< 100 nm	homogeneous surface morphology	Enhanced antifungal and antibiofilm activity against *C.albicans* and *P*.*chrysogenum* compared to PVA alone	[228]
chemical	26 nm	spherical	Reduction of Candidal adhesion and biofilm formation	[229]
Commercial	6 nm	NA	Biocidal against *Aspergillus niger* on Paulownia wood	[230]
Commercial	50 nm	thin homogeneous layer	Antifungal against wood-decaying fungi (*Mucor circinelloides* and *Hypocrea lixii*)	[231]
*Pogostemon cablin*	71.82 nm	NA	Antifungal effect at low MFCs	[232]
Caricaceae (Papaya) shell extracts	15 nm	Semispherical	Antifungal activity against *S. Sclerotiorums* with improved seed germination	[233]
African oil palm	14.60 ± 0.44 nm	agglomerated hemispherical	*Fusarium solani* growth inhibition	[234]
*Trichoderma harzianum*	431 ± 87	spherical	High chitinase activity against *Sclerotinia sclerotiorum*	[139]
*Trianthema portulacastrum* *Chenopodium quinoa*	30–60 nm	Granule-like shapes	Fungicidal effect against *Ustilago tritici*	[83]
ultrasonic	NA	Spherical with aggregation	Inhibited spores’ germination of *F. graminearum*	[235]
*Caesalpinia pulcherrima* flower extract	20–27 nm	spherical	Superior anticandidal activity at low MICs	[236]
**Antiviral**				
Sonochemical method	8 nm	tetragonal	Antiviral treatment of Newcastle disease virus (NDV)	[237]
Chemical hydrolysis	4–10	NA	Viral treatment against H3N2 Influenza virus	[238]
Solid state reaction method	1 m²/g	irregular	Antiviral filtration against H1N1 (face masks)	[239]
chemical impregnation method	NA	amorphous	Antiviral properties against H1N1 and SARS-CoV-2 (COVID-19)	[240]
sol-gel method	50–100 nm	tubular	Potent anti-SARS-CoV-2 activity	[241]
electrochemical anodization technique	10 nm	tubular	Electrochemical sensor for rapid detection of SARS-CoV-2	[242]
Commercial	50 nm	spherical	Antiviral activity against Human Papillomavirus HPV	[243]
Chemical adsorption method	1–100 nm	Anatase	Anti SARS-coV-2	[244]
Sol gel method	20 nm	hemispherical	Antiviral Activity Against Tobacco mosaic virus (TMV) in Pepper plants	[245]
Hydrolysis chemical method	5–6 nm	anatase	Increased silkworm to Bombyx mori nucleopolyhedrovirus (BmNPV)	[246]

#### Antifungal activity

Conventional fungicides and pesticides can be hazardous to health, making nanoparticles (NPs) a more desirable option as they exhibit fewer adverse effects and a more favorable therapeutic index, indicating a better safety margin [247]. Recently, TiO_2_-NPs have revealed fungicidal effects against different human and plant pathogenic fungi such as *Fusarium oxysporum* [248], *Fusarium graminearum* [225], the opportunistic human pathogen *Candida albicans* [226], *Macrophomina phaseolina* [227] (Table 7). Also, the researchers synthesized nanocomposites comprising polyvinyl alcohol (PVA) and TiO_2_-NPs. These nanocomposites were evaluated for their antifungal properties against two fungal strains: *Candida albicans* (ATCC 14053) and *Penicillium chrysogenum* (MTCC 5108); the results demonstrated that the PVA-TiO_2_ nanoparticle biofilms showed enhanced antifungal activity potential comparing to PVA alone. The incorporation of TiO_2_ nanoparticles into the PVA matrix conferred superior antifungal efficacy to the nanocomposite material [228].

**Fig. 5:**
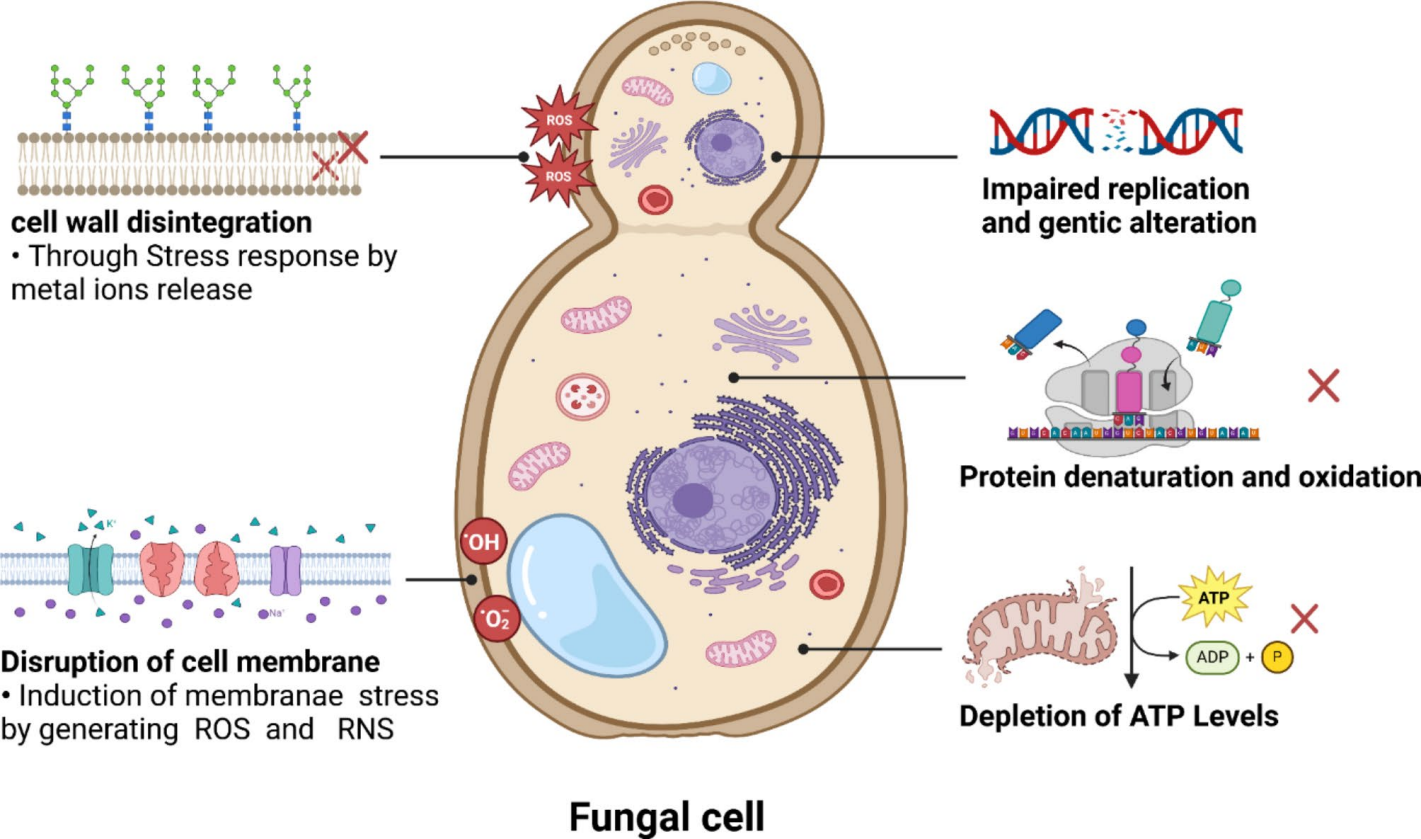
Suggested antifungal mechanisms produced by TiO_2_-NPs

Candidiasis, caused by *Candida* species, is an opportunistic fungal infection affecting various mouth, skin, and genitourinary tracts [249] and can cause life-threatening bloodstream candidemia. It has a high global annual incidence of approximately 4 × 10^6^ cases with a mortality rate of about 40%, resulting in a substantial economic burden due to treatment costs and prolonged hospitalization [250]. It has been reported that metal and their oxide nanoparticles such as (TiO_2_-NPs, Ag-NPs, Cu-NPs, ZnO-NPs) exhibited antifungal activity against *Candida* species through various mechanisms that synergistically contribute to the inhibition and eradication of these fungal pathogens. Firstly, those nanoparticles release ions that disrupt crucial cellular processes within the fungal cells [229]. Additionally, they induce oxidative and nitrosative stress by generating reactive oxygen and nitrogen species, causing deleterious effects on cellular biomolecules such as lipids, and nucleic acids [39]. Furthermore, these nanoparticles can directly interact with and compromise the structural integrity of the fungal cell membrane and cell wall, compromising their barrier functions and potentially causing leakage of cellular contents [251]. Remarkably, certain nanoparticles can inhibit the activity of essential enzymes involved in various metabolic processes, hampering the growth and survival of fungal cells (Fig. 5). Moreover, they can modulate the expression of specific genes involved in stress response, cell wall biosynthesis, and virulence factors, effectively regulating the fungal cell’s ability to thrive [252]. Also, they can deplete ATP levels, depriving the cells of their primary energy currency and impairing cellular functions [253]. Lastly, they can interact with and damage DNA, leading to genetic alterations and impaired replication [254]. They also disrupt protein structure and function and interfere with mitochondrial processes, further contributing to cellular dysfunction and growth inhibition (Fig. 5).

Laboratory and field studies on heritage buildings have also demonstrated the efficacy of TiO_2_ as an effective biocidal agent against lichens and phototrophic microbes, as well as a preventive material against the biodeterioration of buildings [255]. Furthermore, the application of TiO_2_-NPs has been proposed as a protective measure for culturally significant buildings to mitigate the biodeterioration of mortars caused by microbial growth [256]. For example, in one study, researchers evaluated the effects of TiO_2_-NPs against the fungal mold *Aspergillus niger* on the surface of *Paulownia* wood. The results showed that the treatment with TiO_2_-NPs effectively suppressed the growth of the fungal mold [230]. In another study, the antifungal effects of TiO_2_-NPs were assessed against wood-decaying fungal species, including brown rot (*Mucor circinelloides*) and white rot (*Hypocrea lixii*) fungi [231]. This suggests that TiO_2_-NPs possess remarkable antifungal properties and can be employed as an effective protective measure against fungal growth and deterioration in various wood-based applications.

However, it is noteworthy that the antifungal activity exhibited by TiO_2_-NPs appears to be relatively weaker compared to their antibacterial action. This discrepancy can be attributed to the fundamental differences in the structural composition of the cellular envelopes between fungi and bacteria. Fungal cells possess a more robust and complex cell wall structure, which may render them less susceptible to the antifungal mechanisms of TiO_2_-NPs compared to bacterial cells with their relatively simpler cell envelope architecture [257]. Table 7 summarizing examples for antifungal activity of biogenic TiO_2_-NPs against various pathogenic fungi.

#### Antiviral activity

Nanoparticles like Ag, TiO_2_, and carbon nanotubes possess antiviral properties. These properties are employed to showcase the antiviral mechanisms, which include inhibiting the virus from binding to cells and facilitating the breakdown of the viral capsid [258]. Several factors influence the antiviral capabilities of NPs, including their behavior in air and water environments when studied in vitro.

Notably, TiO_2_-NPs have exhibited superior antiviral activities against the Newcastle disease virus (NDV) when assessed at doses ranging from 6.25 to 100 µg/ml (Table 7). The mechanism behind this antiviral activity involves the potential of TiO_2_-NPs to destroy the lipids present in the viral envelope through the generation of ROS, a process known as G-sol. The glycoprotein spikes on the viral surface, which are known to be harmful in facilitating infections, are restricted by an adjunct mechanism, preventing the virus from binding and infecting cells. Consequently, TiO_2_-NPs represent a promising platform for the treatment of Newcastle disease virus infections [237]. Also, TiO_2_ nanostructures were utilized to treat faba bean crops infected with the broad bean stain virus (BBSV). The faba bean plants treated with these NPs demonstrated a significant reduction in the severity of the viral disease compared to untreated plants. This superior antiviral effect was observed within two weeks after the plants were infected with the broad bean stain virus [259].

Further studies have highlighted the antiviral potential of TiO_2_ nanoconjugates and nanoparticles against various influenza viruses. In one investigation, TiO_2_-NPs displayed antiviral capabilities against the H3N2 influenza virus strain, with the proposed mechanism involving direct interaction between the nanoconjugates and the virus [238]. Additionally, TiO_2_-NPs have shown antiviral efficacy against the H9N2, the avian influenza virus. Notably, the replication of two other influenza strains, H5N1 and H1N1, was effectively inhibited by the application of DNA-tagged titanium nitride nanoparticles (TiNPs) [211]. The H1N1 influenza virus, with its high frequency of genetic polymorphism [260, 261] that caused the 2009 pandemic, also posed an increased risk of subsequently developing type 1 diabetes in children infected by it [249]. An interesting study has documented the potent antiviral capability of a TiO_2_-modified hydroxyapatite composite (HA/TiO_2_) against the H1N1 Influenza A Virus when exposed to UV light irradiation. Notably, these composites exhibit potential for antimicrobial filtration applications, rendering them suitable for use in face masks to combat such highly mutable influenza strains [239]. Also, a novel nanocomposite comprising TiO_2_-NPs and polylysine (PL)-containing oligonucleotides, termed TiO_2_⋅PL–DNA. This nanocomposite exhibits antiviral properties against various subtypes of the influenza A virus, including H1N1, H5N1, and H3N2 [240].

Investigations have demonstrated the remarkable disinfection capability of titanium dioxide nanotubes (TiO_2_-NTs) against SARS-CoV-2. These nanostructures exhibited potent anti-SARS-CoV-2 activity at extremely low concentrations in vitro, coupled with negligible cytotoxicity and an insignificant selectivity index (CC_50_/IC_50_ ≤ 1). Moreover, they displayed excellent antiviral efficacy at very low concentrations (IC_50_ = 568.6 ng/mL). Consequently, it was concluded that these TiO_2_ nanostructures are well-suited for use as coatings, serving as potent disinfectants to combat SARS-CoV-2 [241]. While in another study, researchers developed an electrochemical sensor based on TiO_2_- NTs for the rapid detection of SARS-CoV-2. The surface engineering of these TiO_2_ nanostructures has been proposed as a strategy to tailor their potential functionality, enabling real-world applications in combating the SARS-CoV-2 virus [242]. Interestingly, a study explored the potential of TiO_2_ coatings in inactivating SARS-CoV-2 through time-dependent, TiO_2_-mediated photocatalytic reactions. Transmission electron microscopy (TEM) revealed microstructural changes in the SARS-CoV-2 virus upon interaction with the coating. The antiviral activity, assessed in aerosol and liquid forms, exhibited up to 99.9% effectiveness after 20 min of exposure. The mechanistic effects on the SARS-CoV-2 virion included decreased virion count, increased virion size, and reduced particle surface spike structure. Further analyses using western blotting and RT-qPCR investigated the photocatalytic damaging of viral proteins and genomes, respectively. The study concluded that TiO_2_-induced photocatalytic reactions hold promise for disinfecting SARS-CoV-2 and other emerging infectious agents in human habitats [262].

**Fig. 6:**
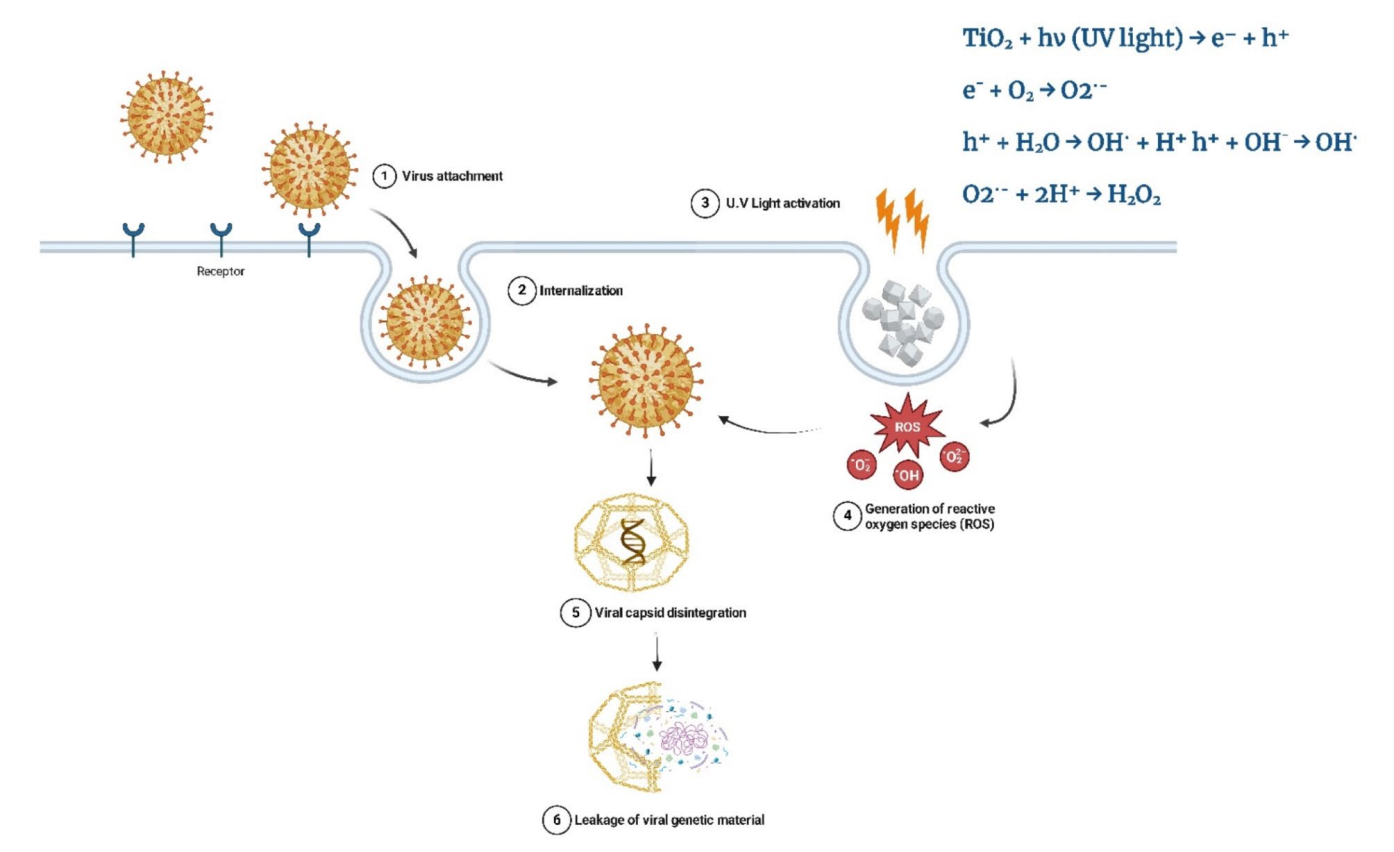
Antiviral mechanisms of TiO_2_-NPs based on secretion of ROS

Under UV light exposure, the TiO_2_ surface facilitates the decomposition of ambient oxygen and water into ROS, which act as highly oxidizing or reducing agents, leading to the decomposition of organic and microbial matter. Furthermore, modified TiO_2_ has demonstrated remarkable utility in visible light activity, enabling indoor or outdoor disinfection applications. This photocatalytic disinfection effect has also shown promising potential as an antiviral photocatalyst for controlling various viruses [211]. ROS, such as OH^•^, O_2_^•-^, and H_2_O_2_, are produced at the surface of TiO_2_ due to UV activation (Fig. 6).

These highly reactive species possess the capability to degrade the capsid proteins of non-enveloped viruses as well as the envelope proteins and phospholipids of enveloped viruses. The degradation of these crucial viral components leads to the leakage of genetic material, resulting in the subsequent degradation of nucleic acids. Consequently, this process culminates in the eventual inactivation of the viral particles, rendering them non-infectious and incapable of replication or causing further infections [263].

### Anticancer activity

Nanotechnology has gained prominence in cancer treatment and diagnosis due to the severe side effects associated with traditional chemotherapeutic agents, which exhibit cytotoxicity on healthy cells. The application of nanotechnology offers a more targeted and precise approach, minimizing harm to normal cells while effectively combating cancer [264]. Nanotechnology research in cancer therapy focuses on developing nanostructures capable of delivering and releasing drugs in a targeted manner. Pursuing targeted drug delivery and controlled drug release represents the primary approach to augmenting therapeutic efficacy while mitigating adverse effects.

TiO_2_ nanostructures, renowned for their high biocompatibility, tunable drug release capabilities, and minimal toxicity, are widely acknowledged as promising candidates for enabling targeted delivery and controlled release of conventional chemotherapeutic agents, consequently enhancing their clinical therapeutic impact [265]. Furthermore, TiO_2_-NPs have demonstrated their efficacy as drug carriers, facilitating targeted delivery of chemotherapeutics like doxorubicin, cisplatin, and temozolomide [266]. The subsequent sections provide a comprehensive review of significant research endeavors that have explored the utilization of TiO_2_ nanostructures in targeted drug delivery mechanisms and diverse controlled release systems, shedding light on their potential in cancer treatment.

#### Target drug delivery

The primary objective of drug delivery systems is to enhance therapeutic efficacy while minimizing adverse effects through appropriate drug encapsulation. These systems have been designed with varying structures, such as spherical, capsular, and porous configurations, to achieve specific drug release profiles [267]. In this context, the drug can be situated on the surface of TiO_2_-NPs, potentially leading to a controlled and sustained release pattern. TiO_2_-NPs are functionalized with specific molecules that possess targeting capabilities toward desired cells, allowing them to localize within tissue-specific cells and thereby enhancing their efficiency. These titanium nanoparticles have been employed as nanocarriers for the delivery of various drugs, including sodium phenytoin, valproic acid, temozolomide, and daunorubicin [268]. Initially, the drug release exhibited a burst pattern, followed by a controlled release over an extended period. Consequently, TiO_2_-NPs demonstrated the ability to deliver drugs with enhanced efficacy, optimized dosing, and controlled and sustained release profiles while simultaneously reducing toxicity levels.

The surface modification of nanocarriers plays a crucial role in enhancing the specificity of drug delivery [269]. A research study explored this concept by modifying TiO_2_-NPs with polyethylene glycol (PEG), followed by the attachment of folic acid (FA) and the standard anticancer drug Paclitaxel (PAC). These anticancer properties were investigated in vivo using a diethylnitrosamine (DEN)-induced hepatocellular cancer animal model. The study’s findings revealed that the surface-modified Paclitaxel (PAC) attached to TiO_2_-NPs exhibited superior anticancer activity compared to PAC alone. Significantly, the surface modification of TiO_2_ with FA in the TiO_2_-PEG-FA-PAC nanocomposite facilitated targeted delivery to liver cancer cells overexpressing FA receptors. This targeted approach resulted in increased accumulation of PAC-NPs at the cancer site, thereby reducing the drug’s toxicity. Moreover, the TiO_2_-PEG-FA-PAC nanocomposite demonstrated a reduction in cell viability correlated with concentration. when tested on HepG2 liver cancer cells, further underscoring its potent anticancer effects [270].

Another study reported the enhanced anticancer activity of FA-TiO_2_-NPs against MG63 osteosarcoma cells. Compared to unconjugated nanoparticles, the FA-TiO_2_-NPs exhibited a two-fold lower IC_50_ value, indicating improved cytotoxicity and efficacy in inhibiting cancer cell growth. The study also explored the apoptosis-inducing effects of FA-TiO_2_-NPs on osteosarcoma cells. Treated cells displayed hallmark apoptotic features: condensed chromatin, surface membrane vesiculation, and cell volume reduction [271].

Remarkably, Annexin V/PI apoptosis assay unveiled a significantly higher percentage (38%) of cells undergoing early and late apoptosis upon FA-TiO_2_-NP exposure compared to only 16% with unmodified TiO_2_-NPs. Moreover, cell cycle analysis revealed an augmented sub-G1 cell cycle in FA-TiO_2_-NP-treated cells, signifying escalated reactive oxygen species (ROS) production and heightened apoptosis induction. A parallel study demonstrated that the delivery of doxorubicin via TiO_2_ nanocomposites facilitated enhanced intracellular drug retention and cellular internalization in multidrug-resistant MCF-7/ADR breast cancer cells, effectively circumventing the P-glycoprotein-mediated efflux mechanism responsible for drug resistance [272]. Researchers in another study developed a pH-responsive drug delivery system by modifying TiO_2_-NPs with hyaluronic acid (HA) and loading them with the chemotherapeutic agent cisplatin for ovarian cancer treatment. This nanoformulation facilitated enhanced cisplatin accumulation within A2780 ovarian cancer cells via endocytosis, exhibiting significant anticancer effects [273].

#### Controlled drug release in cancer therapy

In an effort to enhance therapeutic efficacy and mitigate the undesirable side effects of the chemotherapeutic drug doxorubicin. Doxorubicin was encapsulated within TiO_2_-NPs, forming DOX-TiO_2_-NPs. Subsequent evaluation of the anticancer potential of DOX-TiO_2_-NPs revealed an increased cytotoxic activity against the SMMC-7721 hepatocarcinoma cell line, as demonstrated by the MTT assay [274]. The ratio of Bax/Bcl-2 protein was increased upon DOX-TiO_2_-NP treatment which indicates the endocytosis uptake of doxorubicin, leading to caspase-apoptotic processes [275]. It is worth highlighting that TiO_2_-NPs displayed minimal cytotoxicity when used independently at a concentration of 10 µg·mL^− 1^, with cell viability of 95%, indicating their potential for safe biomedical applications [274].

### Stimuli-controlled drug release

Stimuli-responsive systems are designed to release drugs in response to specific signals. These signals can be classified into two categories: internal and external stimuli. Among the benefits of this system are reducing the side effects of used treatment drugs and increasing the drug’s biocompatibility. They facilitate controlled and tissue-specific drug release, contributing to improved therapeutic outcomes [276]. light emerges as an intriguing external trigger for enabling the controlled and timed release of chemotherapeutic agents from delivery systems, owing to its capacity for precise spatial and temporal targeting [277]. In this context, TiO_2_ nanostructures have garnered substantial interest as photoactive drug carriers. In addition to their photoactive nature, TiO_2_-NPs are preferred as drug carriers due to their characterized high surface area, durability, and accessibility.

In a study, a mesoporous TiO_2_ shell as a core-shell structure for near-infrared light-triggered drug delivery. Doxorubicin was loaded into the porous TiO_2_ shell, and hyaluronic acid (HA) capping was performed. The authors reported that the cell viability was decreased at drug release at low concentrations, indicating its promise for cancer therapy [278]. Also, colloidal TiO_2_-NPs were utilized as carriers for light-controlled delivery of a ruthenium complex drug to melanoma cancer cells. This system exhibited a faster drug release profile upon UV light exposure compared to visible light illumination. Furthermore, cell death increased when exposed to UV light as opposed to red light. The authors proposed that both the TiO_2_-NPs and the ruthenium complex could act as photosensitizers, generating reactive oxygen species and inducing cell death [279]. In another study, DOX was loaded onto TiO_2_-NPs, which were coated with polymeric phenylboronic acid (PBA) through a boronic ester bond. These nanoparticles exhibited high tumor-targeting ability due to the specific interaction between PBA and sialylated epitopes on tumor cells. Additionally, ultrasound irradiation could generate ROS, leading to the release of DOX from the nanoparticles via cleavage of the boronic ester bond [280].

Studies show that increased oxidative stress from elevated tumor ROS levels can be more damaging to cancer cells. ROS generation in the tumor environment triggers desirable apoptotic cell death. Therefore, selectively exposing cancer cells to high ROS levels could serve as a novel target for killing cancer cells without deleterious effects on normal ones [281].

#### Photodynamic therapy of cancer

**Fig. 7:**
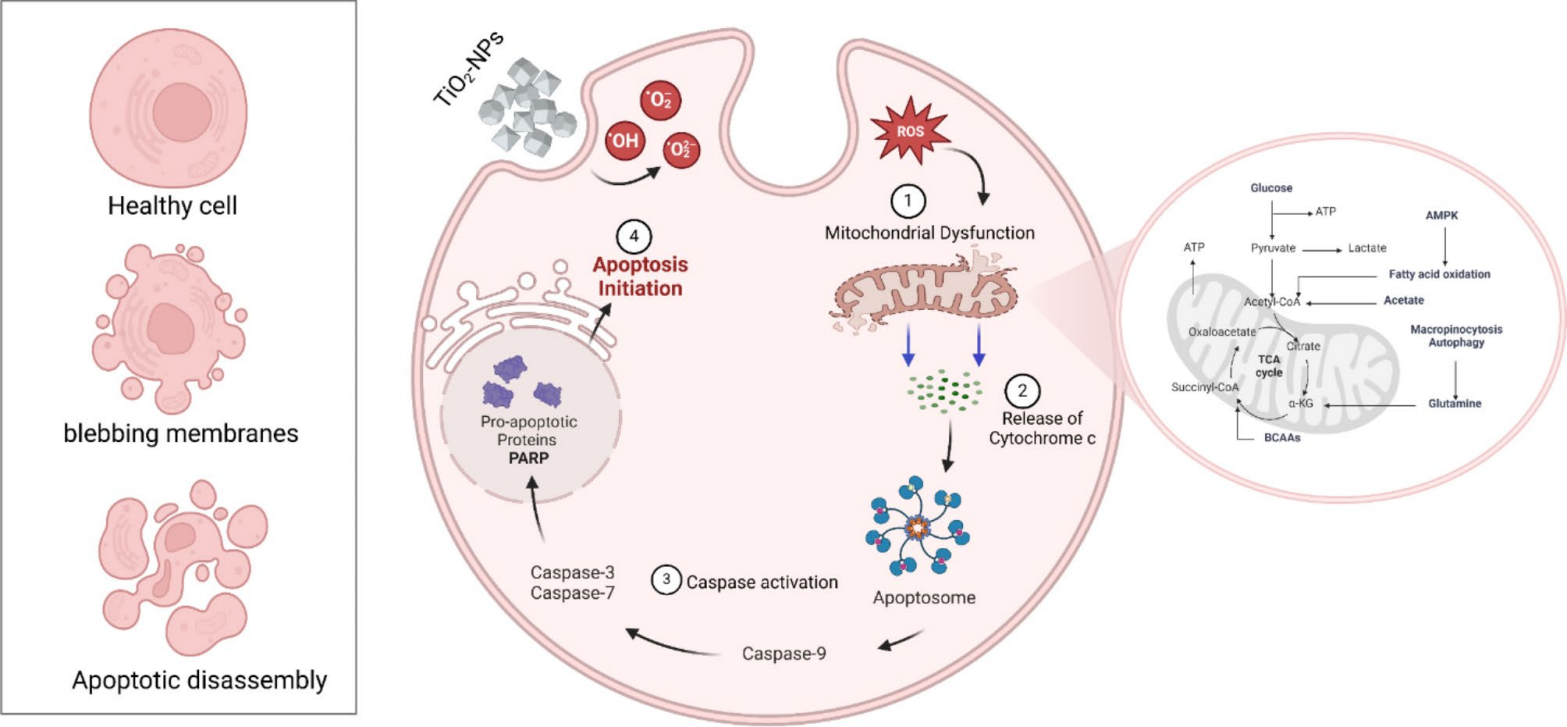
Mitochondrial dysfunction and apoptotic pathways triggered by TiO_2_-NPs

Cancer cells are subjected to laser light to produce a photosensitive agent at a specific wavelength called photodynamic therapy (PDT). Unlike other treatments such as surgery, radiation, and chemotherapy, PDT is considered a secondary and highly promising non-invasive modality for cancer therapy [282]. It offers an encouraging approach as a supplementary treatment option for cancer patients [283]. The inorganic nature of TiO_2_ endows it with the capability to produce ROS when exposed to UV light in aqueous environments (Fig. 7). This ROS generation, which subsequently triggers cell death, positions TiO_2_ as a promising candidate for PDT, a therapeutic approach utilized in the treatment of diverse diseases [284]. The versatility of TiO_2_-NPs, their nanocomposites, and hybrid biomolecular forms has been extensively explored, revealing their potential as photosensitizing agents for cancer treatment and combating antibiotic-resistant bacterial infections [285]. When TiO_2_-NPs are exposed to UV light with a wavelength below 385 nm, photoexcited electrons and holes are generated. Subsequently, these photoexcited electrons and holes can react with OH^−^ or H_2_O, forming oxidative radicals capable of destroying microorganisms and tumor cells (Fig. 7).

The production of ROS by TiO_2_-NPs has been reported to function as effector signaling mediators in the p53-dependent apoptotic pathway. Upregulated expression of cytochrome c, cleaved caspase-3, and PARP, as observed through western blot analysis, further indicated the induction of apoptosis via caspase activation (Fig. 7), highlighting the therapeutic promise of surface-engineered TiO_2_-NPs. The generated ROS can damage the mitochondrial membrane and its functionality, subsequently initiating the mitochondrial release of cytochrome c into the cellular matrix, thereby triggering the intrinsic apoptotic cascade [286].

#### Theragnostic

The convergence of diagnostic and therapeutic approaches has given rise to a new field known as theragnostic. These strategies enable researchers and clinicians to precisely modulate molecular signaling cascades at the molecular level, which is particularly relevant in the context of PDT [287]. In this regard, nanocomposites comprising zinc(II) phthalocyanine (MCZnPc) and MCZnPc anchored onto TiO_2_-NPs labeled with radioactive iodine (125I) were synthesized to evaluate their potential for anticancer applications. The researchers evaluated the efficacy of MCZnPc and MCZnPc-TiO_2_ nanocomposites against HeLa (human cervical cancer) and EMT6 (mouse mammary cancer) cell lines. After a 3-hour incubation period in the dark, the cells were exposed to light at a wavelength of 684 nm. The labeled nanocomposites exhibited enhanced cellular uptake, and the study demonstrated the theragnostic potential of TiO_2_-NPs for cancer treatment [266]. In a recent study, the incorporation of samarium as a dopant into TiO_2_-NPs was shown to enhance the radiosensitivity and cellular toxicity of cancer cells. The doped nanoparticles exhibited increased X-ray absorption, leading to the generation of higher ROS upon irradiation compared to undoped TiO_2_-NPs [288]. TiO_2_-based platforms have been utilized for the detection of small biomolecules, cancer cells, and pathogens in blood samples through various techniques such as label-free microfluidic immunosensors, photoelectrochemical biosensors, field-effect transistors, and amperometric methods [289].

Additionally, Nanoconjugates composed of TiO_2_, polyethyleneimine (PEI), and folic acid (FA) have been developed to construct a controlled drug delivery system regulated by near-infrared (NIR) laser irradiation [290]. The induced and sustained delivery of TiO_2_ nanoparticles facilitated by X-ray exposure can generate electron-vacancy pairs, leading to the structural degradation of organic linkers within the nanoconjugates [291].

### Biosensors

Nanosensors are minute yet highly perceptive devices, with at least one of their sensing dimensions measuring around 100 nm or less. These sophisticated instruments play a crucial role in detecting and evaluating intricate physical and chemical transformations. Moreover, they enable the observation of biochemical and biomolecular alterations within cellular environments, as well as the measurement of hazardous environmental contaminants [292] and optimizing electrochemical biosensors for the precise detection and quantification of toxic chemicals in food products to boost food safety protocols [293]. Biosensors derived from nanomaterials possess remarkable capabilities, enabling highly sensitive and rapid detection of biological entities [294]. The recent fascination with innovative hybrid systems combining biomolecules and TiO_2_ nanostructures has resulted in substantial achievements in manufacturing bio-nano hybrid devices, such as biomolecule-sensitized solar cells (BSSCs) and photoelectrochemical cells (PECs) [295].

In addition, the biosensors field was advanced through the integration of TiO_2_-NPs and biomolecules to form thin films that monitor patients’ responses to medication or surgical treatment [296]. However, for a biosensor to be commercially viable, it must meet specific criteria: cost-effectiveness, user-friendliness, sensitivity and accuracy, rapid response times, and the ability to be manufactured efficiently with high selectivity rates [297].

TiO_2_ nanostructures have been utilized to fabricate a diverse range of sensing devices, encompassing humidity, oxygen, and hydrogen sensors. These nanoscale semiconductors have proven their efficacy as exceptional electrode materials within biosensors, owing to their distinctive properties. Such properties include a porous architecture, providing a vast specific surface area, coupled with outstanding biocompatibility [298]. TiO_2_ possesses the capability to function as an immobilizing matrix, engaging in reactions with the amine and carboxyl groups of enzymes while concurrently preserving their biocatalytic activity [299].

Recently, nanocomposites, including TiO_2_-NPs, are used in biosensors. For instance, nanocomposites containing graphene oxide/TiO_2_-nanowires/chitosan were used to monitor the sequences of specific genes in *Vibrio parahaemolyticus*. The incorporation of TiO_2_ nanowires in the nanocomposite significantly increased the interfacial area, thereby enhancing its sensing capabilities [300]. TiO_2_-based biosensing of target analytes typically employs either electrochemical techniques (such as amperometric and potentiometric processes) or photoelectrochemical (PEC) methods. This alteration manifests as a detectable current signal in the case of amperometric detection or a detectable potential or charge accumulation for potentiometric detection [301]. PEC biosensors, which leverage the photoelectric effect, have emerged as a promising class of electrochemistry-based biosensing devices. These innovative sensors combine the benefits of both optical and electrochemical detection techniques, making them highly attractive for various applications [302].

**Fig. 8:**
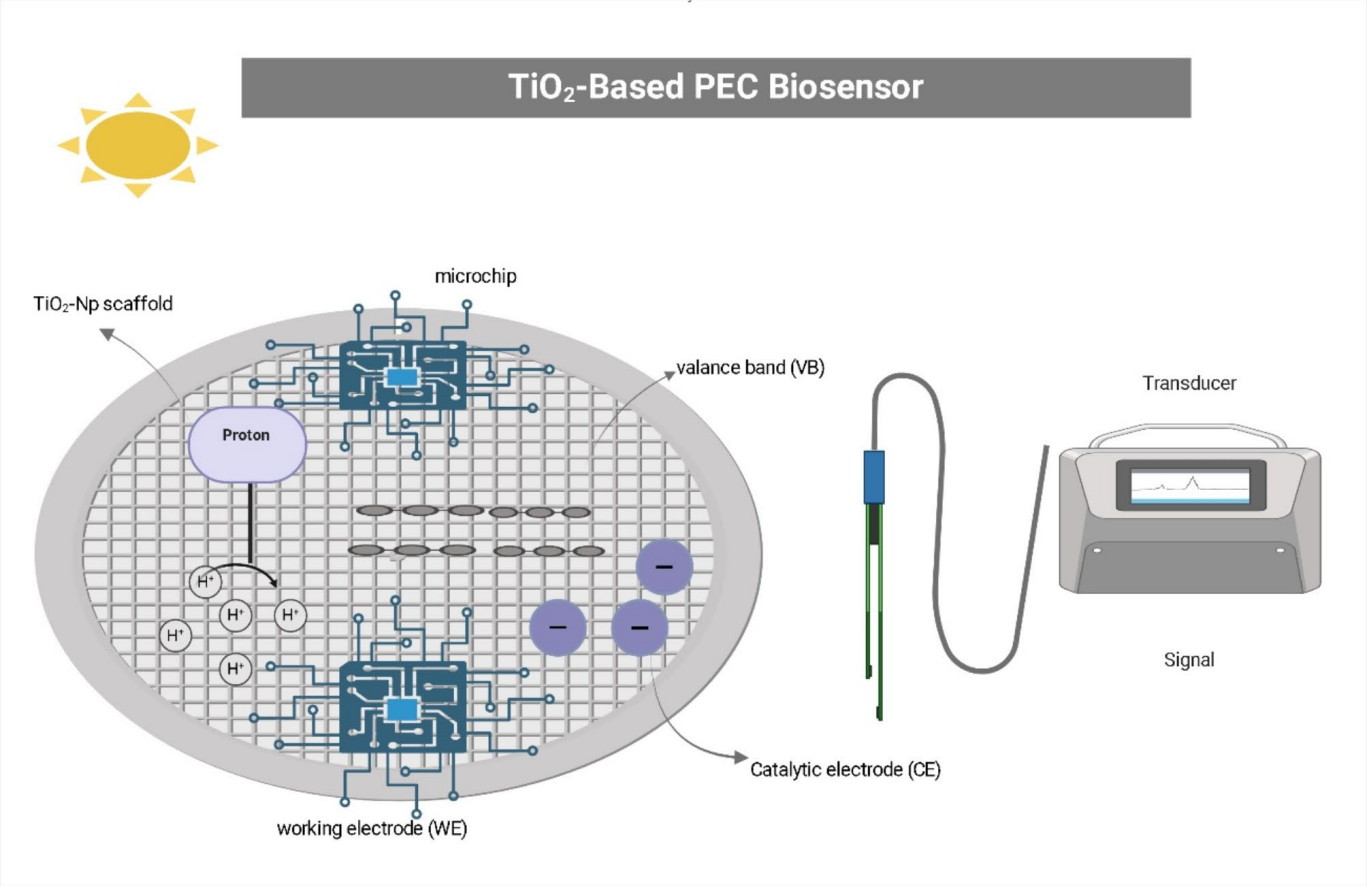
TiO_2_-based photoelectrochemical (PEC) biosensing platform

Typically, a titanium dioxide-based PEC biosensing platform consists of three main components (Fig. 8). Firstly, a nanostructured TiO_2_ layer is fabricated on a conductive surface, acting as the working electrode (WE). This facilitates the generation and movement of electrons and holes upon exposure to light. Secondly, a catalytic counter electrode (CE) coated with an electron transfer material is present. Finally, an electrolyte is placed between the two electrodes, facilitating the shuttling of holes to the counter electrode [296]. In PEC biosensors, the detection principle relies on photon-to-electricity conversion. The process involves photon absorption by the semiconductor, generating electron-hole pairs that separate and transfer to the working electrode and electrolyte, respectively. The presence of the target analyte modulates the photocurrent generated, which correlates directly with the analyte’s concentration in the sample, enabling quantitative biosensing [302]. In TiO_2_-based photoelectrochemical biosensors, the transducer material can take one of three forms: (a) consisting solely of TiO_2_, (b) a hybrid combination of TiO_2_ and inorganic semiconductors, or (c) a composite material where TiO_2_ is combined with other substances.

Diverse biomolecules like enzymes and DNA have served as bio-recognition elements. When DNA is coupled with TiO_2_, the presence of target DNA results in the formation or cleavage of double-stranded DNA, leading to a direct or indirect change in the light-harvesting performance of the working electrode. Consequently, this alteration impacts charge separation and the generated photocurrent [303]. Quantum dots with narrow band gaps can sensitize TiO_2_-NPs and achieve energy band modulation. Cadmium sulfide nanoparticles (CdS-NPs), possessing a narrow band gap of 2.4 eV and a broad excitation spectrum, are well-suited as sensitizing materials. The excitation of CdS -NPs leads to charge separation, facilitating the transfer of conduction band (CB) electrons to the conduction band of TiO_2_. A recent hybrid material combining CdS QDs and TiO_2_-NTs has found application in the sensitive detection of prostate-specific antigen (PSA), a crucial biomarker for prostate cancer [304]. In this application, the CdS QDs were loaded onto the TiO_2_-NTs, which acted as transducers. The sensitization of TiO_2_ with CdS broadens the excitation wavelength range and enhances the photoelectric performance of the TiO_2_ electrodes. Additionally, the coupling between CdS and TiO_2_ reduces the recombination of photo-generated electrons and holes, collectively leading to higher conversion efficiency [305]. When PSA is present, an immune-sandwich assembly facilitates the attachment of an immune-gold-labeled alkaline phosphatase (ALP) to the CdS QDs/TiO_2_ NTs electrode. ALP can catalyze the hydrolysis of ascorbic acid 2-phosphate (AAP) in situ, thereby generating ascorbic acid (AA) for efficient electron donation. The results indicate that an increase in PSA concentration leads to an improved photocurrent response.

PEC biosensors can be combined with alternative detection techniques, enabling the development of biosensors tailored to specific properties and behaviors. In a study, dye-sensitized solar cells (DSSCs) utilizing TiO_2_ nanoparticulate films as the photoanode were integrated with a colorimetric DNA detection approach to sense a particular DNA sequence from Mycobacterium tuberculosis, resulting in a disposable biosensing device [306]. In another study, a nanocomposite consisting of g-C3N4 and TiO_2_ nanosheets was employed to fabricate a photoelectrochemical biosensor capable of detecting glucose with an impressive detection limit of merely 0.01 mM. The two-dimensional TiO_2_ nanosheets, possessing a high specific surface area, demonstrated promising potential for accommodating a substantial glucose oxidase loading. Furthermore, to enable visible light excitation of the photoelectrochemical biosensor and avoid potential deactivation by UV radiation, g-C3N4 was incorporated to minimize the nanocomposite’s bandgap [307].

### Biomedical imaging

There is a wide range of imaging techniques that can be utilized for scientific research and biomedical applications. These include spectroscopy methods like infrared (IR) spectroscopy and Raman spectroscopy, nuclear magnetic resonance imaging (MRI), radio-imaging using specific nuclides, computed tomography (CT) scanning, as well as more advanced scanning techniques such as laser ablation, inductively coupled plasma mass spectrometry (ICP-MS), and matrix-assisted laser desorption/ionization mass spectrometry (MALDI-MS) [308]. Enhancements in diagnostic techniques lead to preliminary treatment and improved recovery prospects for patients. Among nanoparticles, titanium dioxide nanoparticles TiO_2_-NPs are extensively researched and employed in diagnostic methods like MRI and CT scans, serving as contrast agents. Notably, TiO_2_-NPs become activated upon irradiation, enabling them to act concurrently as diagnostic tools and therapeutic agents.

A research study examined the image contrast capabilities of TiO_2_-NPs using magnetic MRI and CT scanners. A clear distinction in imaging was detected between the control samples and those containing TiO_2_-NPs on T2-weighted MRI images. This finding suggests that TiO_2_-NPs could potentially serve as a novel theragnostic agent, offering both radio-sensitizing properties for therapeutic applications and radiological diagnostic functionality due to chemical modifications on their surface [309]. According to the findings, in-depth investigations were carried out on in-situ tagging methodologies for fluorescence microscopy to mark the TiO_2_-NPs internalized by cells. The initial technique involved utilizing fluorescent biotin and fluorescent streptavidin to tag the nanoparticles before and after cellular uptake. Conversely, in the second approach, copper-catalyzed azide-alkyne cycloaddition was employed for labeling and identification of azide-conjugated TiO_2_-NPs. Moreover, synchrotron X-ray fluorescence microscopy (XFM) was utilized to detect TiO_2_-NPs. The results showcasing TiO_2_-NPs by XFM exhibited remarkable alignment with the location of optical fluorescence as detected by confocal microscopy [310].

TiO_2_-NPs can be readily synthesized and modified, such as by incorporating europium(III) ions. Additionally, hollow TiO_2_ nanoshells serve as viable two-photon nanoprobes. When coated with polyethyleneimine, these nanoparticles demonstrate an affinity for binding to HeLa cervical cancer cells, enabling their detection [311]. In another research, a nanostructure comprising a silver core surrounded by a silica shell and an outer mesoporous titania coating (Ag@SiO_2_@mTiO_2_) was developed. The metallic silver core served to enhance fluorescence signals [312].

### Agriculture applications

Contemporary advanced technologies, including aquatic farming, solar-powered greenhouses, genetic engineering, multi-layer crop production, and anti-mold chemicals, have conferred substantial benefits to the agricultural sector by enabling maximum yield production and the cultivation of off-season crops. However, these technologies have also contributed to significant health and environmental concerns due to the improper utilization of fertilizers and pesticides [313]. Nanotechnology is a rapidly evolving interdisciplinary domain focused on developing innovative technological tools to maximize crop yields and enhance plant protection.

The underlying strategy involves boosting plants’ ability to absorb essential nutrients more efficiently. Among various nanomaterials, extensive research efforts have been concentrated on exploring the agricultural applications of TiO_2_-based nanomaterials due to their distinctive structural properties, chemical stability, hydrophilic nature, and environmental compatibility [314]. The subsequent section highlights the applications of nanopesticides and nanofertilizers derived from TiO_2_-NPs.

#### TiO_2_-NPs as nanopesticides

**Fig. 9:**
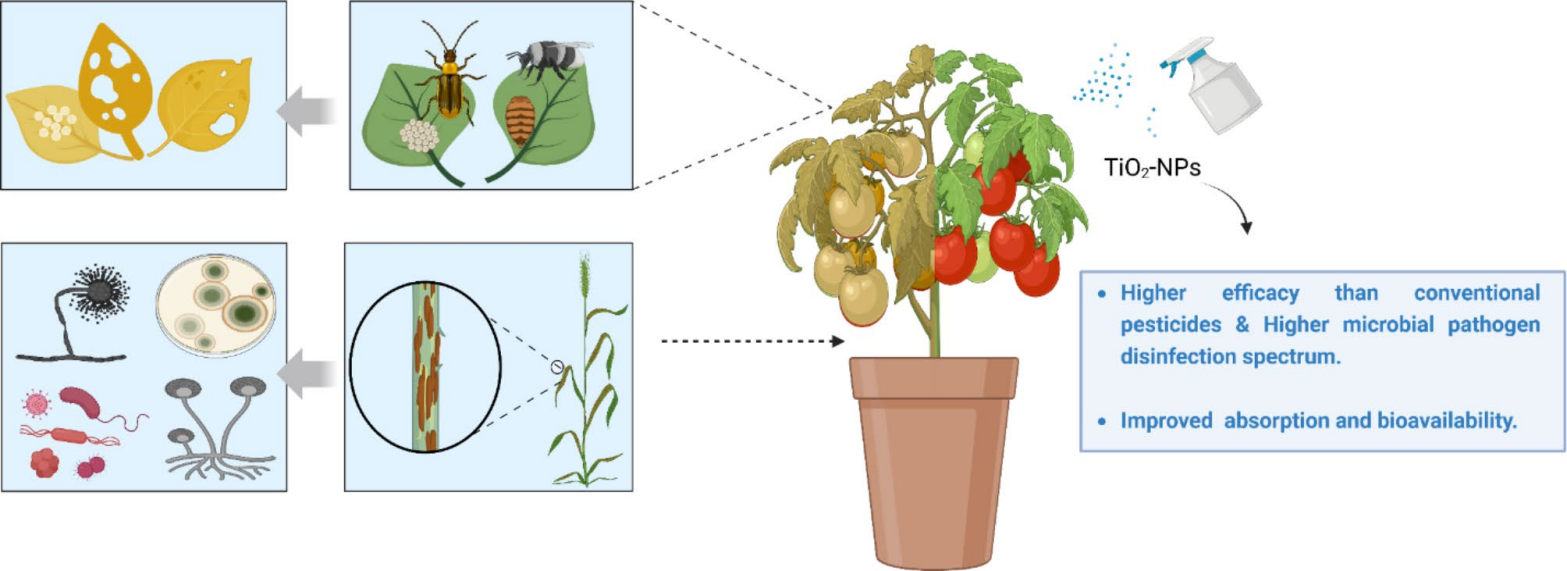
The advantages of utilizing TiO_2_-NPs as nanopesticides for enhancing and improving agricultural yield

Maintaining crop health by preventing diseases and pest infestations is a persistent challenge that drives the creation of novel solutions and agents. Chemical pesticides are employed to manage or eliminate microorganisms, unwanted plants, insects, and fungi. Nevertheless, excessive pesticide usage can lead to severe health implications. Nanoparticles or nanoformulations of pesticides demonstrate greater efficacy compared to conventional pesticides (Fig. 9). This increased effectiveness could be attributed to improved absorption of the active ingredients and higher bioavailability facilitated by NPs, consequently resulting in more efficient elimination of infectious agents [315].

TiO₂-NPs were employed to combat *Spodoptera littoralis*, a polyphagous pest that infests various crops like cotton and vegetables such as tomatoes. An experiment tested six TiO₂-NP concentrations (31.25–1000 ppm) by feeding larvae leaves treated with these nanoparticles. Two weeks after application, mortality was assessed. The findings unequivocally demonstrated the toxic effects of TiO₂-NPs against *S. littoralis* larvae at all the concentrations tested [316]. In another study, TiO₂-NPs were tested individually and in combination with ZnO-NPs to evaluate their insecticidal efficacy against *Bactericera cockerelli* nymphs through laboratory and greenhouse studies on tomato plants. While leaf immersion bioassays were conducted in the laboratory, direct plant spraying was employed in the greenhouse. Results showed that TiO₂- NPs alone (100 ppm) and combined with ZnO -NPs (250 ppm) caused 99% and 100% mortality, respectively, after 96 h of treatment in the laboratory. However, in the greenhouse experiment, TiO₂- NPs (500 ppm) and TiO₂ + ZnO NPs (250 ppm) resulted in only 32% and 23% mortality, respectively [317]. Also, a different study explored the insecticidal potential of TiO₂-NPs prepared via a green synthesis method using the aqueous leaf extract of *Pouteria campechiana*. The findings revealed that at a concentration of 900 µg/ml, the larvae and pupa of *Aedes aegypti* were dead [318].

TiO_2_-NPs loaded with fluorine and nitrogen were used to inhibit the *Fusarium oxysporum* growth in tomatoes under visible light conditions by destroying of fungal cell wall. The synergistic effects of TiO₂-NPs and the attached N & F resulted in a stronger toxic impact on the fungal strain, ultimately leading to its eradication. The generation of ROS under visible light illumination enabled the disinfection of the fungus. Consequently, these TiO₂-NPs could be utilized for visible light-induced bacterial and fungal disinfection [248]. In another investigation, the antibacterial capabilities of TiO₂-NPs and TiO₂-NPs doped with silver (Ag) and zinc (Zn) showed high antibacterial activity (in-vitro and greenhouse experiment) against *Xanthomonas perforans* that causing tomatoes spot disease at a concentration of 500–800 ppm under visible light conditions. This enhanced activity was attributed to the combinatorial effects of TiO₂ and the Ag and Zn dopants [319]. Table 8 summarizes the activity of TiO_2_-NPs in agricultural sectors as nanopesticides.

**Table 8:** Examples of TiO_2_-NPs used in the agricultural sector to control the infection with different pests, insects, and microbes

Nanoparticles treatment	Synthesized TiO_2_-NPs source	Target	Host	Results	Reference
TiO₂-NPs	Leaf aqueous extract of *Pouteria campechiana*	*Aedes aegypti* (larvae and pupa)	Not detected	Maximum death of larvae and pupa was attained at 900 µg/ml	[318]
TiO₂-NPs	Commercial with concentrations of 31.25–1000 ppm	Tested against *Spodoptera littoralis* larvae	Cotton, tomatoes	Toxic effects at all concentrations	[316]
TiO₂-NPs + ZnO-NPs	Chemical TiO₂ (100 ppm), ZnO (250 ppm)	*Bactericera cockerelli* nymphs	Tomato plants	Causing 99% mortality under lab conditions and 32% mortality under greenhouse conditions	[317]
TiO₂-NPs (co-doped fluorine and nitrogen)	Not detected	*Fusarium oxysporum*	Tomatoes	Completely eradicated fungal strain under visible-light condition	[248]
TiO₂-NPs + Ag/Zn dopants	Commercial with concentration of 500–800 ppm	*Xanthomonas perforans*	Tomatoes	High antibacterial activity under visible light	[319]
TiO₂-NPs	*Desmostachya bipinnata*	*Spodoptera litura* and *Aedes aegypti* (larvae and pupa)	Not detected	The highest mortality of 96% and 94% against *A. aegypti* and *S. litura* respectively was attained at 900 µg/mL.	[320]
	*Solanum trilobatum*	*pediculocidal*, and *larvicidal activities*	*A subpictus* and *Hyalomma anatolicum*	High mortality rate at 2–10 µg/mL of TiO_2_-NPs	[321]
	*Beauveria bassiana*	Noctuidae pests	*Helicoverpa armigera* and *Spodoptera frugiperda*	50% mortality of caterpillars with lower toxicity	[322]
	*Moringa oleifera*	*Bipolaris sorokiniana* infection	wheat	40 mg/L of TiO_2_-Np was sufficient to reduce disease severity	[323]
	*Bacillus thuringiensis*	*Ephestia kuehniella* larvae	Mediterranean flour moth	Potential nano pesticides, 74% larval mortality	[324]
Ag/Fe/TiO_2_-NPs	*Trichoderma harzianum*	*Sclerotinia sclerotiorum*	*Anodontites trapesialis*	Fungicidal action after 48 of TiO_2_-Np exposure	[325]

#### TiO_2_-NPs as nanofertilizers

**Fig. 10:**
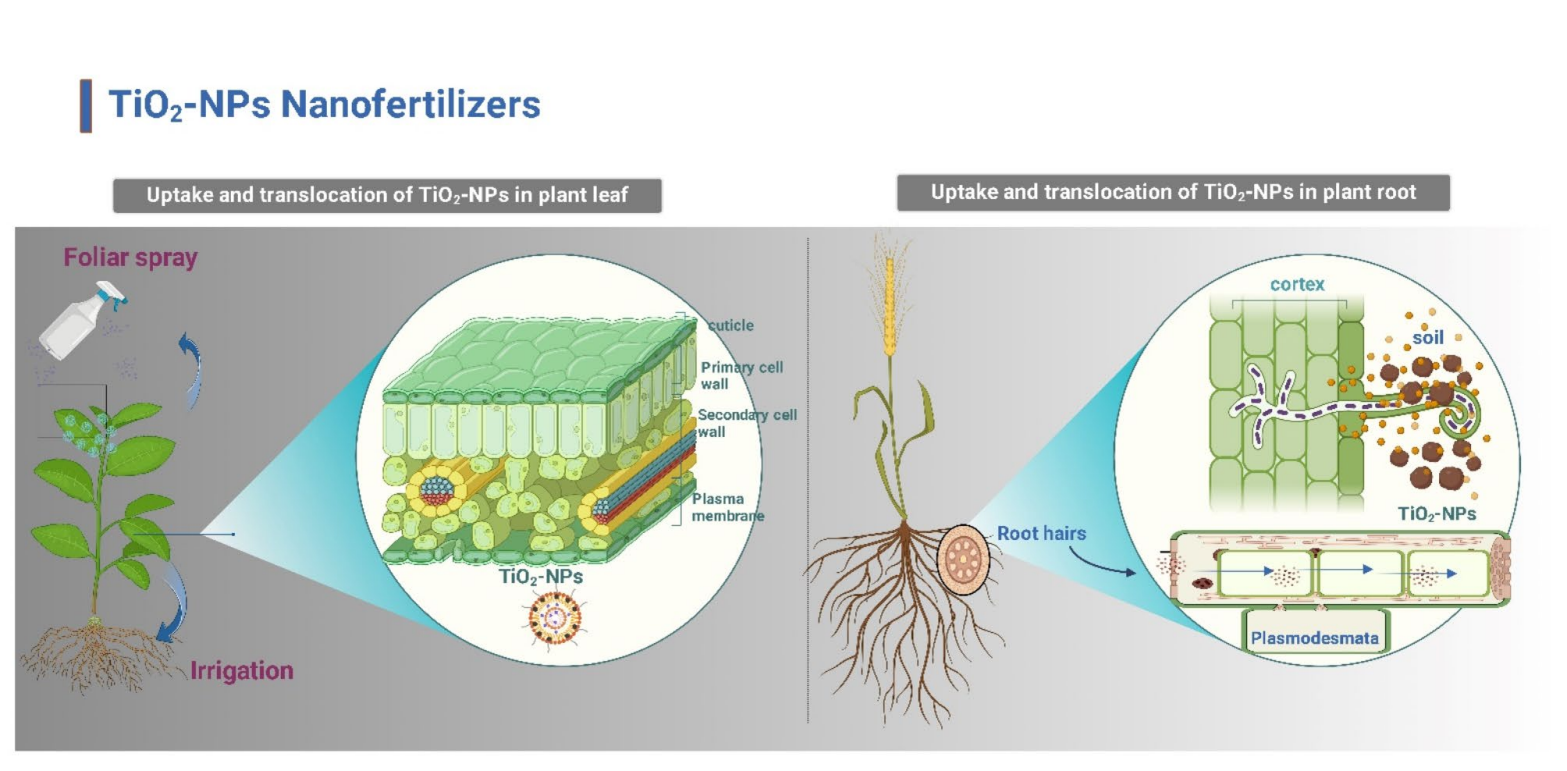
Foliar spray and soil irrigation routes of TiO_2_-NPs as nanofertilizers

**Fig. 11:**
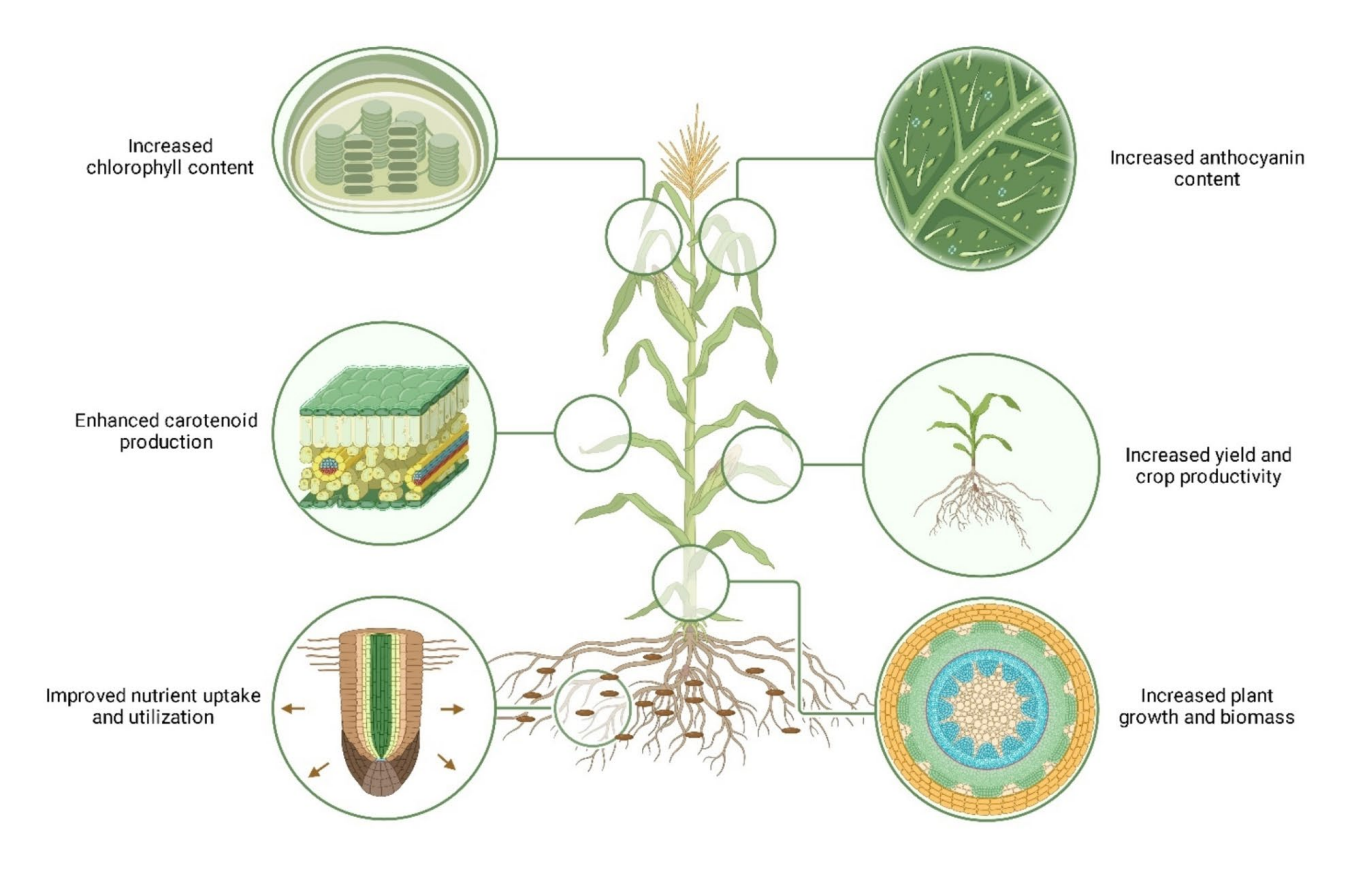
Advantages of utilizing TiO_2_-NPs as nanofertilizers to improve plant growth traits

The incorporation of fertilizers can boost agricultural productivity. However, to address issues such as environmental pollution and inefficient nutrient utilization, nano fertilizers emerge as a preferable option, potentially serving as a more effective and efficient alternative to conventional fertilizers. Additionally, nano fertilizers contribute to enhancing soil quality by mitigating the detrimental effects associated with the excessive application of conventional fertilizers [326]. TiO_2_-NPs can be utilized in the context of nanofertilizers via diverse approaches, such as foliar administration, seed pretreatment involving immersion, and soil hydroponic methods where TiO_2_-NPs are incorporated into the irrigation solution or combined with soil substrates [327]. Foliar spraying is a prevalent method for facilitating rapid absorption of TiO_2_-NPs through stomatal uptake and cuticle layer penetration. Their transport through cell walls and distribution within the plant vascular system is facilitated by their size and surface traits. Conversely, soil applications are frequently chosen due to its prolonged nutrient release capability. TiO_2_-NPs are internalized by root hairs across the root epidermis, moving symplastically via plasmodesmata between cells, and subsequently transported upwards through stem vascular tissue to the leaves (Fig. 10).contributing to elevated chlorophyll levels, augmented photosynthetic efficiency, and consequently enhanced plant biomass and yield (Fig. 11).

The choice of application technique hinges on the specific crop, its development phase, and intended objectives. Foliar spraying is prevalent for its direct delivery of nanoparticles to photosynthetic tissues, while soil application is favored for prolonged nutrient accessibility.

The influence of TiO₂-NPs, a nano fertilizer, was studied on spinach seedlings during their growth and development stages. The treatment with TiO₂-NPs resulted in improved seed vigor and germination rates. Furthermore, at a 2.5% concentration of nanoTiO_2_, enhancements were observed in plant dry weight, Rubisco activity, chlorophyll formation, and phytosynthetic rate during the growth phase [328].

Mahmoodzadeh and coauthors investigate the effects of TiO_2_-NPs on the seedling vigor of canola plants [329]. The authors reported that the seedling and radical growth of canola was increased at high TiO_2_-NPs concentration (2000 mg/L). In a related investigation, the plant growth (vegetative parts, male and female flower appearance) and phytosynthetic pigment content (chlorophyll, anthocyanins, and carotenoids) of *Zea mays* were affected by spraying of TiO_2_-NPs at various TiO₂-NPs concentrations. The results showed that the 0.03% TiO₂-NPs concentration had a notable effect on chlorophyll a and b, carotenoids, total chlorophyll, and anthocyanins. Additionally, the pigmentation during the reproductive stage was improved upon TiO₂-NPs spray as compared with control. These activities could be due to the positive impact of TiO₂-NPs on the electron transport chain and photosynthesis processes, thereby boosting pigment production [330].

Recently, TiO_2_-NPs formed by fruit peel extract of *Citrus medica* were used to improve the *Capsicum annuum* yield [331]. Also, the foliar spray of TiO_2_-NPs on sunflowers at a concentration of 2.6 mg/L led to improved physiological traits and nutritional parameters and increased the content of oil [332]. Interestingly, the height of coriander plants, as well as their physiological traits, carotenoid contents, total sugars, amino acids, and phenols, were improved as a result of spraying with TiO_2_-NPs at 2, 4, and 6 ppm [333]. These studies collectively demonstrate the potential of TiO₂-NPs to enhance various aspects of plant growth and development across varied species, highlighting their promising applications in agriculture (Table 9).

**Table 9:** Summarizing the activity of TiO_2_-NPs in agricultural sectors as nanofertilizers

Synthesized by	TiO₂-NPs Conc. and type of treatment	Results	Reference
Spinach seedlings	2.50%, seeds soaking	Improved seed vigor, germination rates, plant dry weight, Rubisco activity, chlorophyll formation, photosynthetic rate	[328]
Canola	2000 mg/L, seeds treatment	Increased seedling and radical growth	[329]
*Zea mays*	0.03%, spraying	Notable effects on chlorophyll a and b, carotenoids, total chlorophyll, anthocyanins, improved pigmentation during the reproductive stage	[330]
*Capsicum annuum*	Not detected	Enhanced plant growth	[331]
Sunflowers	2.6 mg/L, foliar spray	Improved physiological traits, nutritional parameters, increased oil content	[332]
Coriander	2, 4, and 6 ppm, spraying	Improved height, physiological traits, carotenoid contents, total sugars, amino acids, phenols	[333]
Wheat	25–100 µg/mL, foliar spray	Reduced salinity and improved germination with 50 µg/mL TiO₂ -NPs	[87]
Rice	1000 mg/L, root exposure	Stress tolerance at lower conc.	[334]
bell pepper	250 mg/L, leaves spray	Increased disease resistance	[335]
Rice	750 mg kg^− 1^, irrigation	Boosted crop yield and improved rhizosphere enzymatic activity	[336]
Moldavian balm	200 mg/L, added to nutrient solution	Increased volatile oil content under salt conditions	[337]
*Vitex* plant *Vitex agnus-castus* L.	0-800 ppm, foliar spray	Higher sugar content and boosted shoot and root dry weight mass	[338]

### Environmental applications

Natural resources such as water, air, and soil are severely affected worldwide, and their recovery is difficult due to rapid population growth, urbanization, and industrialization. Several reasons lead to water and soil contamination. The main ones are the direct release of raw industrial effluents without treatment into rivers and sewage management, which interferes with the indiscriminate use of pesticides and fertilizers in agriculture parties. Presently, the pollution of our environment is so widespread that it has turned into a critical issue. Water and soil are contaminated with harmful heavy metals, chlorinated compounds, or dyes. At the same time, the atmosphere is filled with noxious nitrogen oxides (NO), carbon monoxide (CO), volatile organic compounds, and chlorofluorocarbons (CFCs). Given the high levels of contamination, now is the time to employ advanced technology for monitoring and identifying these pollutants in our water and soil [339].

Nanotechnology has experienced remarkable advancements in the field of environmental protection over the recent years [340]. Among its most promising contributions are the significant applications in water and air remediation. The nano-size, surface area to volume ratio, chemical NPs stability, surface modifications, and shapes are considered the unique features of NPs, making them superior activity for environmental applications either in-situ or ex-situ [341]. Different types of available nanomaterials and nanotools are being utilized to remediate environmental contaminants [342]. Among the various materials, utilizing TiO₂-NPs as a remediating agent is witnessing a steady rise in applications such as water purification, air cleaning, and soil decontamination. The electronic band structure, high quantum efficiency, stability, and chemical inertness for TiO_2_-NPs enable them to be resilient and adaptable to diverse conditions and uses, especially for contaminant removal [343]. Table 10 summarizes the activity of TiO_2_-NPs in removing soil and water contaminants.

**Table 10:** Examples of different TiO_2_-NPs and their rule in soil and water remediation

Contaminants	Treatment	Results	Reference
Diphenyl arsenic acid (DPAA)	TiO_2_-NPs	DPAA removal efficiency up to 82.7%; adsorption of inorganic arsenic byproducts	[344]
p-Nitrophenol	TiO₂-NPs combined with pulsed discharge plasma	Removal of p-nitrophenol up to 88% within 10 min	[345]
Cadmium (Cd)	TiO₂-NPs combined with plant growth-promoting rhizobacteria (PGPR)	Enhanced Cd uptake, increased chlorophyll content, and promoted *Trifolium repens* seedlings	[346]
Antimony (Sb)	TiO₂-NPs combined with biochar	Increased Sb accumulation and improve *Sorghum bicolor* seedlings	[347]
lead (Pb)	TiO₂-NPs adsorbents (0.1 g) using surfactants	97% of pb(ii) ions removal from contaminated soil	[348]
Cadmium (Cd)	TiO₂-NPs synthesized by *Trianthema portulacastrum* and *Chenopodium quinoa*	Efficient cd removal from industrial wastewater	[349]
Cu²⁺ and Rhodamine B (Rh-B)	TiO₂-NPs synthesized by *Chlorella vulgaris*	High contaminants removal within 1 h.	[350]
Industrial textile dyes, methylene blue (MB) and Rh-B	TiO₂-NPs synthesized by *durva* herb	High degradation activity of MB and Rh-B days after 50 min	[351]
Organic contaminants	TiO₂/Arabic gum	Degradation of ciprofloxacin and MB	[352]
Phenols	TiO_2_/Algae Complex	98% phenol degradation after nearly 19 h	[353]
Methylene blue	TiO₂-NPs synthesized by mulberry plant	MB disintegration within 2 h	[354]
Organic Dyes (RB19 &RR76)	TiO₂-NPs synthesized by *Eichhornia crassipes*	0.08 g of TiO_2_-NPs in 60 min. under U.V at PH = 1 achieved complete degradation	[355]

#### Soil remediation

Soil contamination occurs due to the presence of hazardous compounds, particularly heavy metals at toxic levels. The sources of soil pollution include manufacturing activities, mining operations, and landfill sites containing industrial wastes like paint residues, electrical wastes, batteries, and industrial or municipal sewage [356]. Heavy metals pose a significant challenge as soil pollutants because they are non-degradable and persist in the environment once introduced [357]. Numerous research studies have revealed intriguing findings pertaining to the use of TiO₂-NPs for soil remediation through the UV-facilitated degradation of organic contaminants present in the soil [358].

The photodegradation of diphenyl arsenic acid (DPAA), a pollutant resulting from leakage of arsenic weapons with adverse health effects, has been studied using TiO_2_-NPs. Various operational parameters like NP dosage, radiation time, light intensity, and soil-water ratio were optimized to enhance DPAA removal efficiency up to 82.7%. While TiO₂-NPs don’t completely convert DPAA, they adsorb its inorganic arsenic byproducts, playing a crucial role in mitigating DPAA contamination through photocatalytic oxidation [344]. Another study investigated the photocatalytic degradation of p-nitrophenol, a bio-refractory and toxic organic compound widely used in various industries, from contaminated soil using TiO_2_-NPs combined with pulsed discharge plasma. The mechanism involved removing p-nitrophenol from the soil and enhancing its degradation by increasing the pulsed discharge voltage. The authors proposed that the pulsed discharge plasma could drive the photocatalysis of TiO_2_-NPs. The report revealed that this approach could remove up to 88% of p-nitrophenol within just 10 min [345].

Also, a study examined the combined application of plant growth-promoting rhizobacteria (PGPR) and TiO_2_-NPs for enhancing phytoremediation of cadmium (Cd) contaminated soil using *Trifolium repens* seedlings. The soil had 28% clay, 37% sand, 35% silt, 0.47% N, 7.1 mg/kg phosphorus, and pH 7.8. Different doses of TiO_2_-NPs and PGPR were applied separately and in combination to analyze their effects on Cd uptake, plant growth, and chlorophyll content. The combined application promoted plant growth, increased chlorophyll content, reduced the required TiO_2_-NPs dosage for phytoremediation, and enhanced Cd uptake and *T. repens* growth in Cd-contaminated soil compared to individual applications. This highlights the potential synergistic effects of PGPR and TiO_2_-NPs for efficient phytoremediation of heavy metal-polluted soils [346]. The combined application of biochar and TiO_2_-NPs was explored for phytoremediation of antimony (Sb) contaminated soil using *Sorghum bicolor* seedlings. The soil had a pH of 7.7, 1.12% N, 8.7 mg/kg phosphorus, 28% clay, 37% sand, and 35% silt. Different concentrations of TiO_2_ and biochar were applied individually and in combination to assess their effects on plant growth, Sb uptake and accumulation, and physiological responses in Sb-polluted soil. The results showed that the combined biochar and TiO_2_-NPs treatment positively influenced plant growth and significantly increased Sb accumulation compared to individual applications. The findings demonstrated the potential of this approach for efficient phytoremediation of heavy metal-contaminated soils [347].

#### Water remediation

**Fig. 12:**
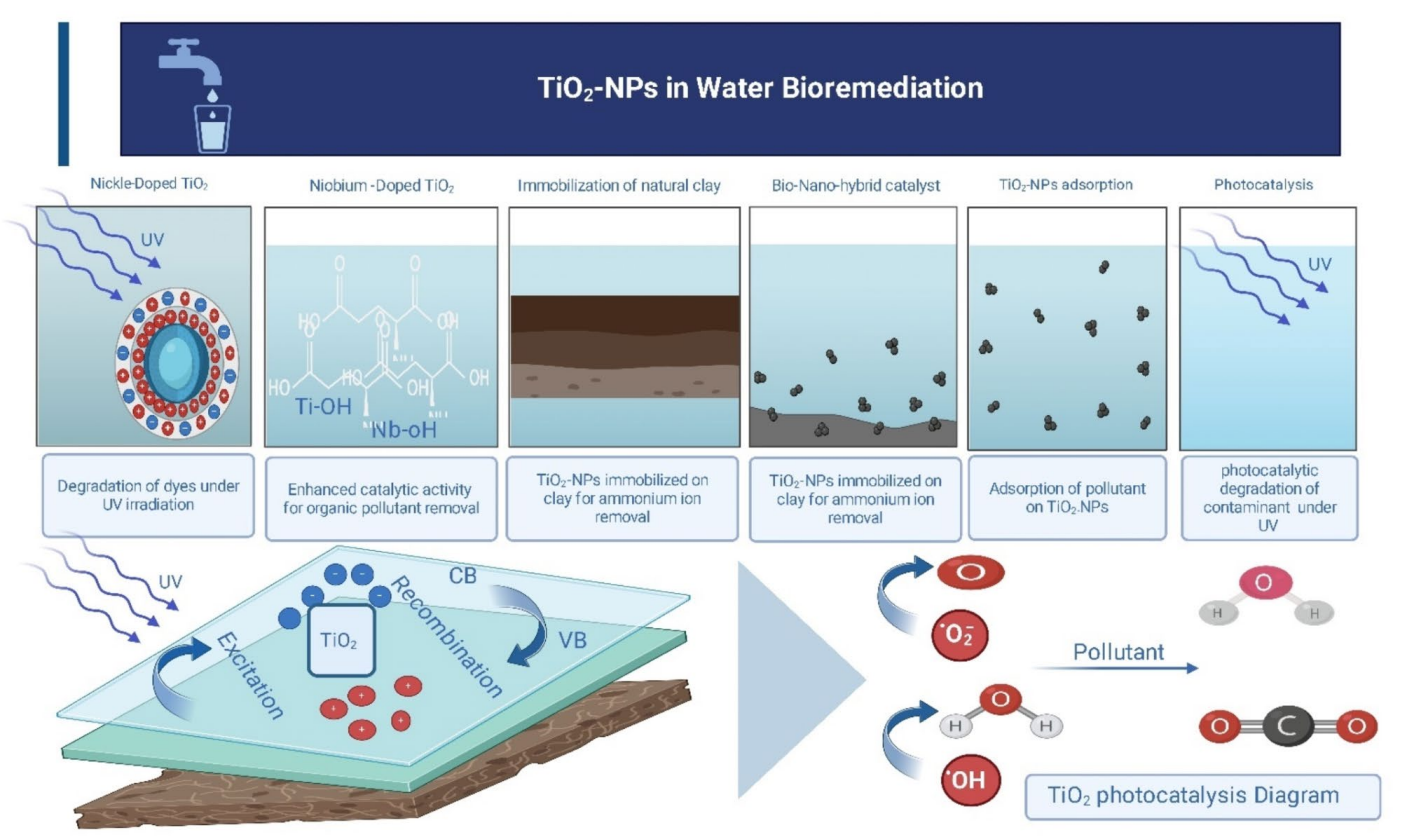
Enhanced photocatalytic activity of TiO_2_-NPs in organic pollutants degradation and their role in water remediation

Clean and fresh water is a vital necessity for daily life and the life cycle of living organisms. Recently, water resources have been highly contaminated with a wide range of pollutants as a result of human activities [359]. Among these contaminants are physical (such as impurities that alter the water’s physical features), chemical (such as heavy metals and organic and inorganic pollutants), and biological pollutants. These pollutants have negative and deleterious impacts on humans, animals, and the ecosystem [360]. Therefore, the treatment of wastewater based on nanoscience has been given more attention. Adsorption and photocatalytic-based nanotechnology are among the approaches for wastewater treatment that have gained a lot of traction due to their eco-friendliness, sustainability, and cost-effectiveness. Additionally, they offer various other advantageous properties that make them particularly suitable for tackling the complex issue of water contamination [361]. In this regard, TiO_2_-NPs are widely employed in water remediation (Fig. 12) due to their unique physical and chemical features, highly biocompatible, robust oxidation efficiency, and unparalleled photocatalytic properties [10]. This prominence has led to a surge in research focused on harnessing the potential of TiO₂-NPs for wastewater treatment, with numerous studies exploring their efficacy in this domain.

## Conclusion

In summary, this review underscores the pivotal role of TiO_2_-NPs in nanotechnology. The principal findings point out that TiO_2_-NPs showcase notable versatility and potential across diverse sectors, including biomedical research, agricultural advancement, and environmental restoration. Their distinctive characteristics, such as high surface area and photoactivation customize them for various medical and ecological applications, including targeted drug delivery, photodynamic therapy, and environmental decontamination. The insights above emphasize the need for continued exploration of bio-based synthetic techniques and detailed bioactivity mechanisms, focusing on enhancing their efficacy and safety in healthcare applications and improving their usage in agronomic practices.

## Future perspectives

The future of TiO_2_-NPs holds immense potential, with numerous exciting avenues awaiting exploration. By addressing current challenges, leveraging cutting-edge technologies, and fostering interdisciplinary collaborations, researchers can unlock the full potential of these remarkable nanoparticles, driving innovation and advancing scientific frontiers across various domains.

Synthesis Advancements: The green and biogenic synthesis routes for TiO_2_-NPs have gained traction owing to their eco-friendly nature and sustainability. However, there is significant scope for optimization and scaling up these biological synthesis methods to achieve industrial-scale production while maintaining monodispersity and precise control over nanoparticle size, shape, and crystallinity. Integrating cutting-edge techniques, such as machine learning and computational modeling, could accelerate the design and development of novel biogenic synthesis strategies, enabling the tailored production of TiO_2_-NPs for specific applications.

Surface modifications and hybrid nanostructures: The surface chemistry of TiO_2_-NPs plays a pivotal role in determining their functionality and performance. Future research endeavors could focus on developing innovative surface modification strategies, including doping, functionalization, and incorporating biomolecules or polymers. These approaches can potentially enhance the nanoparticles’ biocompatibility, stability, and targeted delivery capabilities, expanding their applications in biomedicine, sensing, and catalysis domains.

Moreover, integrating TiO_2_-NPs with other nanomaterials, such as graphene, carbon nanotubes, or quantum dots, could give rise to hybrid nanostructures with synergistic properties. These hybrid systems may exhibit superior performance in areas like photocatalysis, energy conversion, and optoelectronics, paving the way for developing advanced technologies and devices.

Theragnostic applications: The remarkable photocatalytic and optical properties of TiO_2_-NPs have enabled their exploration in biomedical applications, including cancer therapy, antimicrobial treatments, and bioimaging. Future research could delve into developing multifunctional TiO_2_-based nanoplatforms that seamlessly integrate diagnostic and therapeutic capabilities. Such theragnostic systems could facilitate early disease detection, targeted drug delivery, and real-time monitoring of therapeutic responses, ushering in a new era of personalized and precision medicine.

Environmental remediation: The photocatalytic activity of TiO_2_-NPs has demonstrated promising potential in environmental remediation applications, such as water purification, air cleaning, and soil decontamination. Future efforts could focus on enhancing the photocatalytic efficiency and visible-light absorption of TiO_2_-NPs through doping, surface modifications, or developing hybrid nanostructures. Additionally, integrating TiO_2_-NPs into sustainable and scalable technologies, such as membrane filtration systems or photocatalytic reactors, could facilitate widespread adoption in environmental remediation processes.

Interdisciplinary Collaborations: The multifaceted nature of TiO_2_-NPs necessitates multidisciplinary collaborations among researchers from diverse fields, including chemistry, materials science, biology, medicine, and environmental engineering. Such collaborative efforts foster cross-pollination of ideas, enable the exchange of knowledge and expertise, and accelerate the translation of fundamental research into practical applications.

## Data Availability

No datasets were generated or analysed during the current study.
